# AFG - Active Faults Greece: a comprehensive geomorphology-based 1:25,000 fault database

**DOI:** 10.1038/s41597-025-06283-z

**Published:** 2025-11-21

**Authors:** John G. Begg, Vasiliki Mouslopoulou, David Heron, Andy Nicol

**Affiliations:** 1 National Observatory of Athens, Institute of Geodynamics, Athens, Greece; 2 GNS Science, Lower Hutt, New Zealand; 3 School of Earth and Environment, University of Canterbury, Christchurch, New Zealand

**Keywords:** Tectonics, Natural hazards, Geology

## Abstract

Greece is Europe’s most seismically active country, as it is being deformed by an active subduction-system and one of the world’s fastest-spreading continental rifts. Onshore active faults pose seismic-hazard that cannot be reliably assessed in the absence of a comprehensive map of potential earthquake sources. Here, we use high-resolution Digital Elevation Models (DEMs), in conjunction with hillshades and slope-models, to map and characterise faults in Greece at a scale of 1:25000. The Active Faults Greece (AFG) database records 3815 fault-traces assigned to 892 interpreted faults. Of these traces, 53% were mapped here for the first time, with their geometries and slip-sense constrained by displacement of landscape features. AFG includes >2000 active and 1632 probably-active traces, while 35 traces result from historic surface-ruptures. Many faults (57%) exhibit strong depositional-control (DC) on sedimentation patterns, with active faults featuring approximately equal numbers of sharp (32%), moderate (29%) and rounded (29%) scarps. AFG is the first fault database in Greece generated using nationwide interpretation of geomorphology, with applications in paleoseismology, seismic-hazard-assessment, mineral-resources exploration and resilience-planning.

## Background & Summary

Seismic hazard in tectonically active regions like Greece is strongly influenced by the distribution, geometry and activity of crustal faults. Accurate mapping of fault traces is a prerequisite for estimating credible earthquake magnitudes on individual faults and developing a national seismic hazard model. Historical earthquakes that have ruptured the ground-surface globally provide information on the dimensions, slip patterns and timing of future events^1–4^. These earthquakes afford an empirical basis for estimating the likelihood of rupture termination at fault irregularities (e.g., steps and bends)^5^, and for assessing the conditions that favour co-seismic transfer of slip between faults (e.g.^6,7^). Historical surface-rupturing earthquakes in Greece have also highlighted the occurrence of temporally clustered slip on nearby faults (i.e., 1981 M6.4-6.7 Alkyonides Earthquake Sequence^8^), of multi-fault ruptures (i.e., the 1978 Thessaloniki Earthquake^9^), and of earthquakes that ruptured concealed faults or faults with subtle geomorphological expression (e.g. the 2008 M6.7 Ilia-Achaia Earthquake^10^ and/or the 2021 Tyrnavos Earthquake Sequence^11^). Multi-fault ruptures have the potential to produce larger and less frequent earthquakes, with greater total slip than events restricted to individual faults (i.e.^12–14^), while rupture of concealed and/or sub-resolution faults can be expected for all fault systems^15^, and may have important repercussions for seismic hazard, as was the case for the 1987 Edgecumbe earthquake^16^ and the doublet of Christchurch earthquakes in New Zealand^17^. Thus, where possible, subtle and/or concealed traces should be incorporated into fault maps and seismic hazard models.

Despite experiencing hundreds of historical moderate to large-magnitude damaging earthquakes (i.e.^18,19^), to date Greece lacks a landscape-based active fault map of national coverage. Three active fault databases which are publicly available (GreDaSS, NOAFaults, HeDBAF)^20–22^, have been developed or are being developed from a selection of existing literature and unpublished data. Here, we build a new open-access homogeneous database of continental active faults in Greece: The **Active Faults Greece** (**AFG**) database. AFG represents a development in active-fault mapping for Greece, both in terms of its coverage and methodological consistency. It includes all previously known active faults with appropriate referencing, revisited here using geomorphic criteria on high-resolution DEMs, as well as numerous newly identified structures, and effectively doubles the number of fault-traces of existing national compilations.

AFG comprises active faults, probably active faults, and faults with uncertain Quaternary activity, all constrained by interpretation of geomorphic features in the landscape. These features include fault scarps, displaced terraces, alluvial fans, ridge crests, facetted spurs, linear valleys and range fronts, which serve as diagnostic indicators of neotectonic activity and help establish relative rates of fault activity. This approach enhances the interpretability, reliability, and future adaptability of the database, especially in regions where paleoseismic or geophysical data are limited. The value of this approach derives from its national extent, uniformity of compilation methods and a consistent philosophy of geomorphic attribution. Central to this active-fault compilation was to redraft all previously described faults and map new faults using landscape features, observed on high-resolution DEMs (2 or 5 m grids) of national extent, during a process of careful and systematic scanning. As a result, we remapped the surface traces of 1783 previously recognised fault-traces (in many cases significantly modifying previously reported fault-trace locations, geometries and lengths) and introduced 2031 new fault-traces. Our database displays active-fault geometries, activity and geomorphic expression at 1:25,000 scale, always with reference to previously published work. Our mapping philosophy emphasizes a unified and reproducible methodology that integrates high-resolution topographic data (e.g., DEMs, satellite imagery, hillshades and slope maps), remote sensing imagery, field observations, and, where applicable, sub-surface datasets (e.g. trenching & seismic-reflection lines). This strategy was designed to address ambiguities and inconsistencies between existing Greek fault databases, which often differ in scope, purpose, and inclusion criteria.

Beyond its scientific rigor, AFG serves broader goals: it offers a resource for understanding and managing Greece’s earthquake risk. AFG provides a foundation for national and regional seismic hazard models, supports earthquake preparedness and resilience planning, and offers a reproducible model for other nations seeking to systematize active fault data collection and presentation. Last, the 2023 Mw 7.8 and 7.7 Kahramanmaraş earthquakes in southeastern Turkey — triggered by a cascade of fault ruptures^13,23^, including previously quiescent segments—underscore the urgent need for active-fault datasets that not only reflect known seismicity and ‘easily identifiable’ faults on the landscape but also anticipate potential sources of future events on subtle or quiescence segments.

## Methods

Faults form within the Earth’s brittle crust and, the largest of them, are capable of generating M > 6 earthquakes, with the seismic energy released causing ground shaking over distances up to hundreds of kilometres from their epicentre. Fault-surfaces are commonly buried beneath the ground surface and are generally visible only in outcrops (i.e.^24^), and/or through the use of geophysical tools^25^. Fault ruptures during large-magnitude (>M6) earthquakes often, however, propagate to the ground-surface and displace topography (e.g. fan surfaces, spurs, ridge-crests and streams), with displacements ranging from a few cm’s to several metres (i.e.^26–28^). It is these fault traces that comprise the core of the AFG in onshore Greece.

**Fig. 1:**
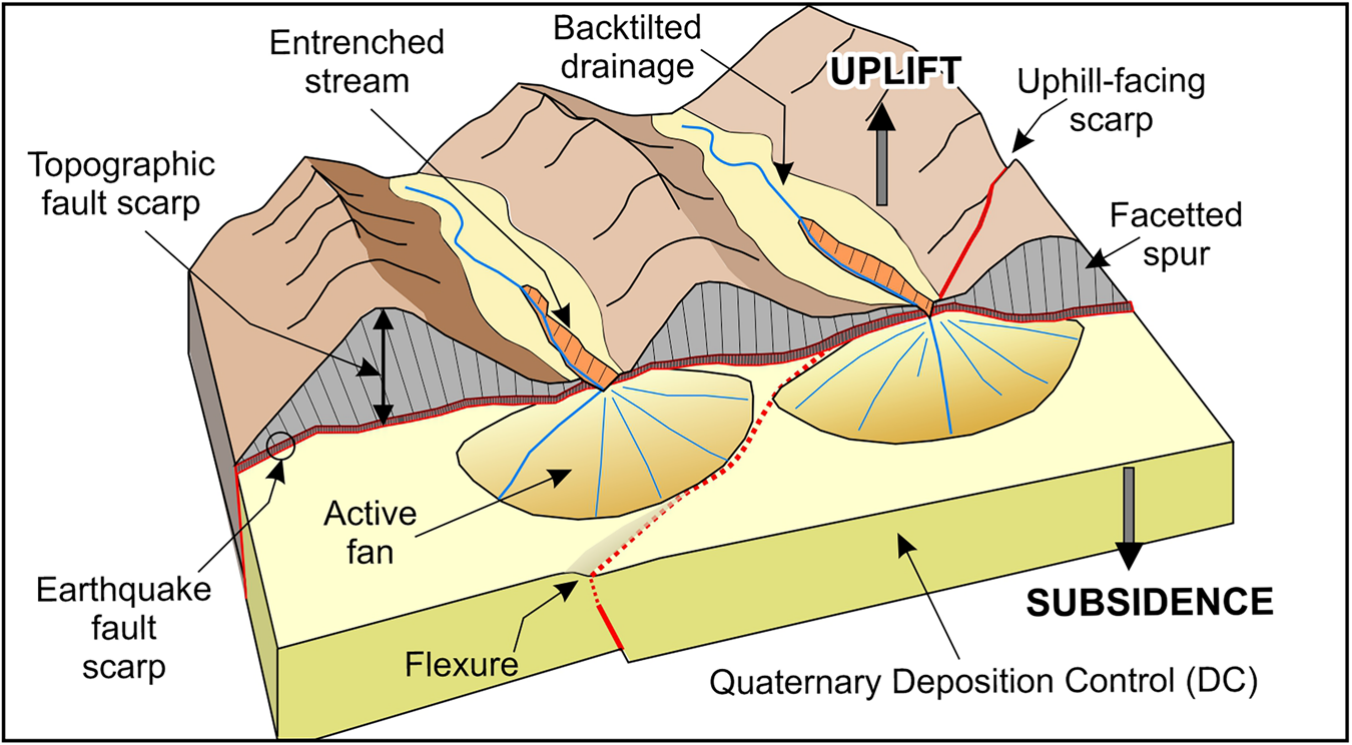
Summary of landscape features used in AFG. Schematic diagram illustrating the main geomorphic features used to trace the surface expression of active faults in AFG (such as earthquake fault-scarps, topographic fault-scarps, uphill-facing scarps, flexures, etc) atop a schematic Quaternary depocenter (DC).

**Fig. 2:**
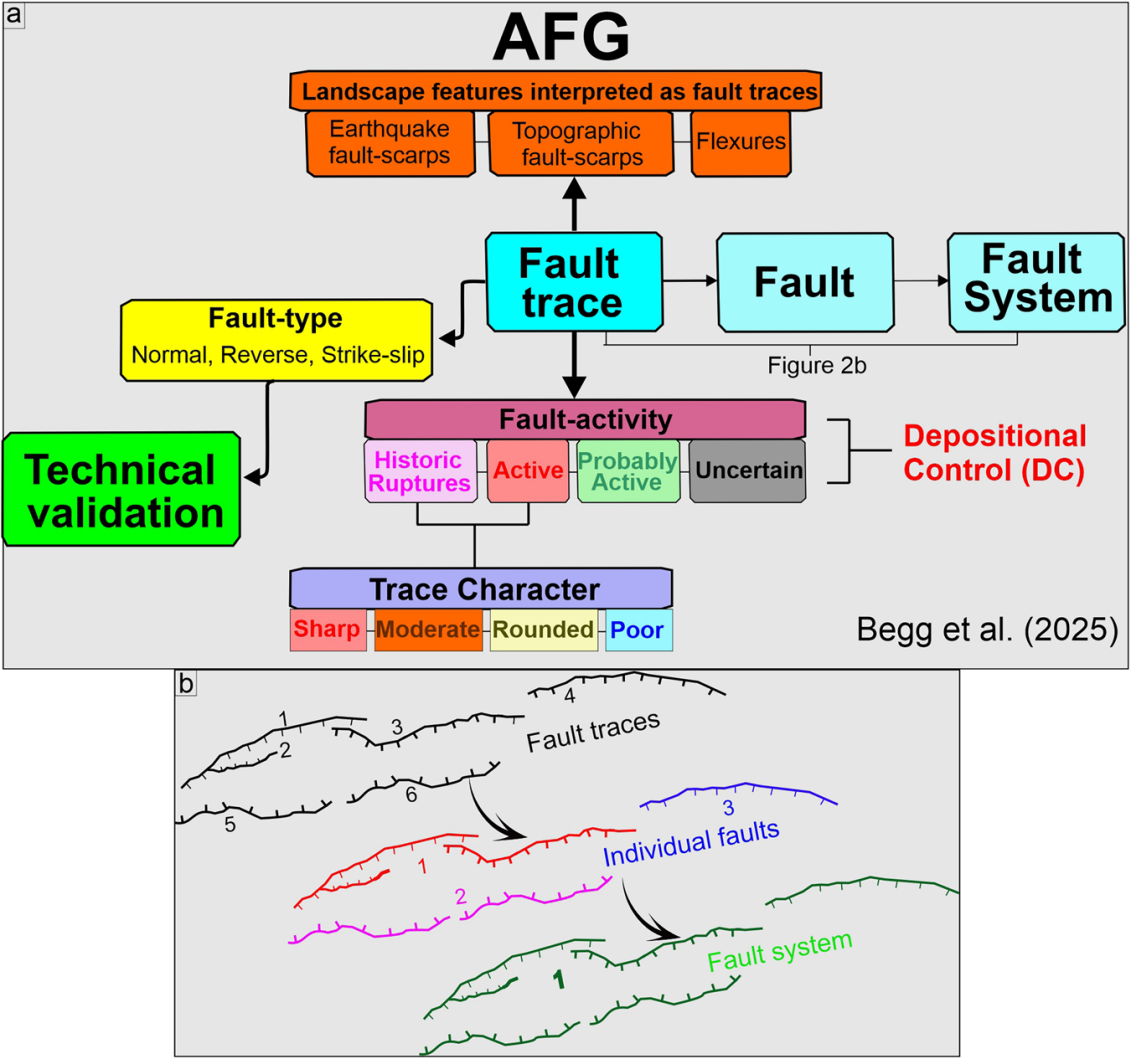
(**a**) Flow chart summarizing the AFG structure. (**b**) diagram illustrating schematically the upscaling in AFG from individual fault traces to faults and, where applicable, fault systems.

In the following, we first present our methodological approach to mapping individual fault-traces using a number of specific geomorphic criteria (see ‘*Fault trace mapping*’ section) and subsequently discuss the individual steps taken to characterise and attribute each AFG trace. Collectively, the AFG contains 3815 normal, reverse and strike-slip fault traces (see *‘Classification of fault type’*), with each trace represented on the landscape as earthquake fault-scarp, topographic fault-scarp or flexure (Fig. 1). Fault traces are subsequently merged into individual faults and, where possible, fault systems (see section ‘*Fault Hierarchy: from fault traces, to faults and fault systems*’) (Fig. 2a,b). By tailoring the definition of active faulting for Greece (see discussion below), fault traces are subsequently classified as historically active, active, probably active or of uncertain activity. Last, following specific geomorphic criteria, active fault traces are classified as sharp, moderate, rounded or poor (see section ‘*A geomorphic classification in AFG*’). The flow-chart in Fig. 2a summarizes the methodology developed in AFG and discussed in detail below.

### Fault trace mapping

The AFG is of Greek national onshore coverage and has been compiled at 1:25,000 scale. Because it is geomorphologically-based, it is restricted to fault traces with surface expression (with the single exception of a concealed fault that ruptured historically and was identified through its aftershock sequence^10,29^). Linear landscape features such as ridge-crest damage, ridge-crest displacement, valley alignment, air-gaps along ridgelines, or drainage displacements were used as proxy indicators for faulting^30^. Initial systematic geomorphological scans of DEMs, hillshades and slope-maps allowed identification of lineaments that warrant further investigation. To assess the tectonic origin of these lineaments we tested them against the following geomorphic criteria, with the requirement that at least one is honoured (Fig. 1a):

- Measurable displacement of materials traversed by traces or lineaments.
- Prescence of fault facets or facetted spurs.
- Stream-channel displacement or changes in channel-incision across the inferred trace.
- Linear range fronts adjacent to areas of active deposition.
- Elongate slope changes that cut geomorphic features and cross geological units.
- Elongate lineaments clearly distinct from basement texture and/or cross-cutting bedrock fabric.
- Significant displacement or truncation of ridge-lines.

**Fig. 3:**
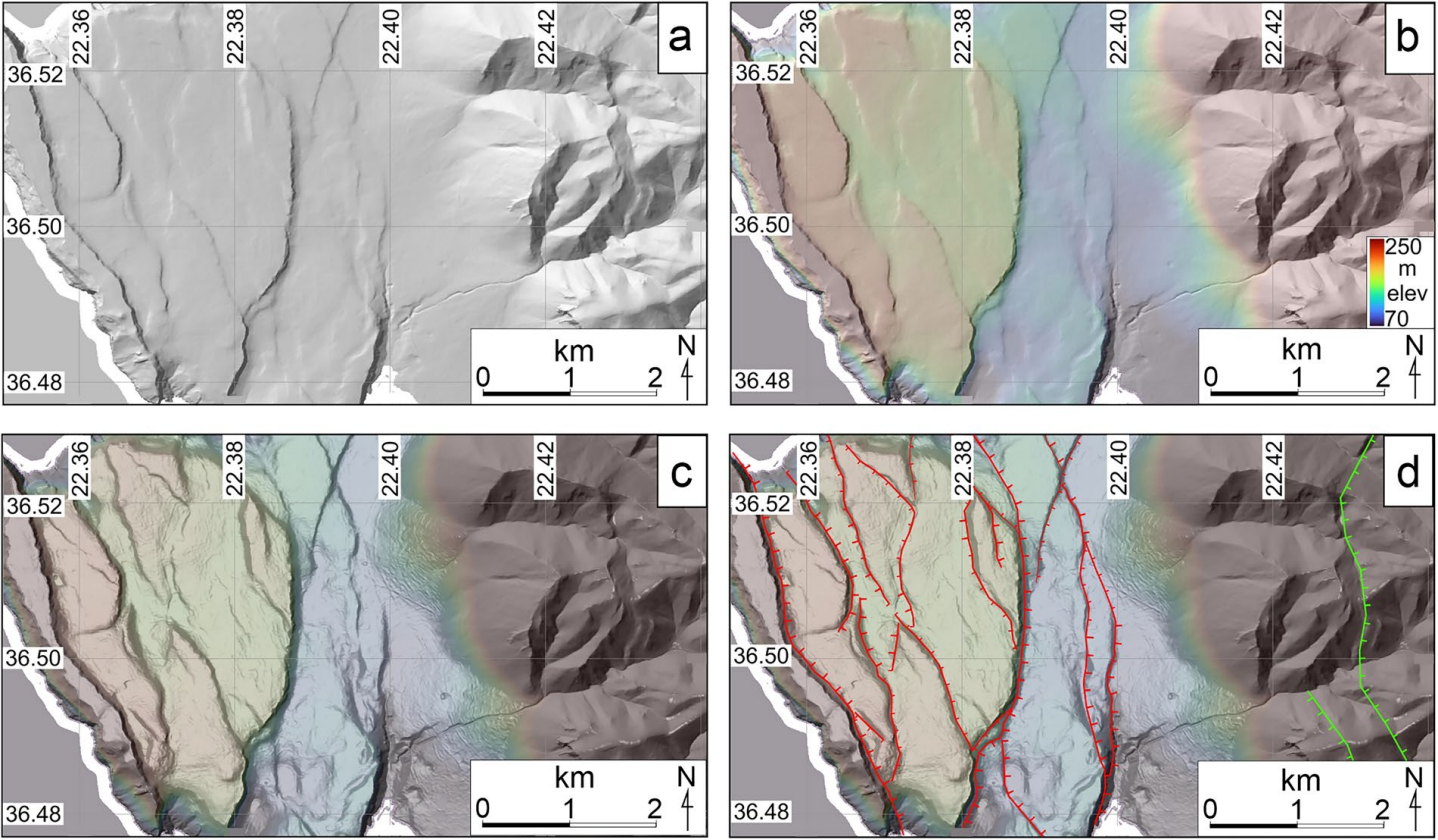
Enhanced fault mapping through digital elevation raster images. Figure demonstrating the methodology followed to map fault traces using digital elevation models and various derivative raster images. Here, an area immediately NW of Gerolimenas in south Peloponnese is (**a**) lit from 315° at 40°; (**b**) illustrated with slightly transparent hillshade (70% transparency) underlain by the colour-ramped DEM; (**c**) is shown as colour ramped DEM and hillshade overlain by slightly transparent (70%) slopemap; and (**d**) populated by fault traces mapped using collectively the above data.

Here, the term displacement refers to any type of displacement (vertical, horizontal, or oblique), and the above criteria apply to dip-slip (normal or reverse), strike-slip (left or right-lateral) and/or oblique-slip faults. Following verification that these features represented fault traces, digitisation and attribution of geometric fields (dip direction and orientation) followed. All assembly and processing of data included in AFG were completed using the open-access software QGIS^31^. Tiles of 2 m digital elevation models (DEMs) from the Greek Cadastre Agency were merged prior to fault mapping, and hillshades from varying azimuths of illumination and inclination angles were generated and used as overlays on colour-ramped DEMs. Derivative slopemaps were also used as overlays and proved particularly useful in identifying low, linear traces across gently sloping landscapes (e.g. Figure 3). Existing published literature describing and mapping active faults in Greece was assembled and all relevant maps were georeferenced. Each fault was checked against published georeferenced maps to determine whether it had previously been identified. Where previously identified (even approximately), appropriate citation was included. Thus, an empty shapefile, with a coordinate reference system of WGS 84 (EPSG:4326), was progressively populated with digitised and attributed lines during a complete and systematic scanning of the entire country. Google Earth was used, where possible, to corroborate the presence of faults using satellite imagery.

It should be noted that there are several places where fault traces are discontinuous in geomorphic expression over distances of a kilometre or more (i.e. where Holocene deposition obscures pre-existing scarps) and thus the trace cannot be mapped at 1:25,000 scale within these intervals. In these cases, fault-traces are located as accurately as possible within the context of the landscape between mappable traces. Further, where lineaments are parallel to bedrock fabric, including bedding, they have commonly not been interpreted as fault traces in AFG. Last, short traces (length <100 m) with little or no displacement, particularly when extending proximal to other larger features (i.e. fault-splays at tips or fault junctions), have not been mapped exhaustively. Thus, while the suite of AFG traces identified from the DEMs is not exhaustive, those present are believed to represent all major faults and fault systems in onshore Greece that outcrop at the ground surface.

### Classification of fault type

Observed displacements of landscape features underpinned assignation of a ‘fault type’ for each AFG trace (normal, reverse or strike-slip). Dip-slip on landscape features can be readily observed where fault scarps are formed and record vertical displacement. Almost all traces in AFG are attributed to being elements of normal faults or fault systems (Table 1). Assessing whether there has been oblique or strike-slip movement on a fault is more difficult and requires careful examination of the landscape to identify lateral displacements of unambiguous piercing points (e.g., ridge-crests and spurs, or streams). For a fault with long-term downthrow on the hanging-wall resulting in active deposition, strike-slip displacement may be difficult to observe in the landscape. However, laterally offset fans and stream courses should demonstrate strike-slip displacement. Despite careful examination for indicators of significant lateral displacements, only 20 strike-slip traces were recorded (Table 1), suggesting that strike-slip displacement is unlikely to be an important deformation mechanism at the ground surface in onshore Greece.

**Table 1:** Table summarizing the characteristics of the fault traces included in the AFG database.

Fault Trace Attributes		
Total number of fault traces	3815	
Total number of faults	892	
Total number of fault systems	237	
**Faults by Activity**		
Αctive faults	2090	55%
Probably active faults	1626	43%
Historic fault-trace ruptures	35	1%
Uncertain faults	64	2%
**New and Previously Mapped Fault Traces/Faults**		
New fault traces	2031	53%
Previously mapped fault traces	1783	47%
**Fault Traces by Type**		
Normal	3798	>99%
Reverse	2	<1%
Strike-slip (dextral or sinistral)	20	<1%
**Fault Length**		
Minimum fault length (km)	0.5	
Maximum fault length (km)	161.5	
Average fault length (km)	16.6	
Median (m)	11.5	
**Geomorphic Characteristics of Active faults**		
Sharp fault traces	708	34%
Moderate fault traces	595	28%
Rounded fault traces	590	28%
Poor fault traces	198	9%
Faults characterised	**2091**	
**Depositional Influence**		
Depositional control (DC)	2167	57%
Non Depositional Control (na)	1651	43%

### Earthquake fault-scarps, topographic fault-scarps and flexures

The 3815 AFG traces mapped using the DEMs include earthquake fault-scarps, topographic fault-scarps and flexures (Fig. 1a). Fault-scarps represent surface displacements along active faults produced either by single surface-rupturing earthquakes (co-seismic fault scarp) or, more commonly, due to several (<10) large-magnitude earthquakes on individual faults^32,33^. Earthquake fault-scarps are sharp and often linear or gently curved discontinuities commonly of <20 m height. In limestone bedrock these scarps are defined by the outcropping fault surfaces, which typically dip at 60–70°. Fault-scarps due to historic surface-rupturing earthquakes in Greece range in dip-slip from 10 cm to 4 m (*i.e*.^34–36^). In contrast, topographic fault-scarps are larger topographic lineaments that represent 100 m to kilometer-scale displacements accrued on faults over 100’s of thousand to millions of years due to many (10s to 1000s) large-magnitude earthquakes^32,37^ (Fig. 1a). Flexures recorded from the DEMs may also result from a series of rupture events that, in this case, failed to penetrate to the ground surface (Fig. 1a). Here, the location of the buried fault may be deduced from an approximately linear fold developed in the existing landscape^30,38^. The majority of active faults recorded in AFG are earthquake and/or topographic fault-scarps (Fig. 1a).

### Fault hierarchy: from fault-traces to faults and fault systems

Active faults commonly include numerous individual fault traces. Although fault traces may or may not be linked in map-view, they usually merge at depth to form a common slip surface that represent a single coherent active fault (*i.e*.^38,39^). This is often the case in AFG, where neighbouring normal fault-traces are interpreted to represent a single fault at depth (Fig. 1b). In such cases, we upscale, using a different attribute-field, these line observations from ‘trace’ to ‘fault’ (Table 1; Fig. 2a,b). We have used one or more criteria to determine which fault traces are part of an individual fault. These criteria are: i) fault traces are approximately co-linear with a common dip direction, ii) fault traces are formed along a single continuous range-front, and iii) fault traces are closely spaced (<3 km horizontal separation). Similarly, where individual faults are located proximal to one another and appear to accommodate displacements interdependently^40^, we have upscaled the ‘fault’ interpretation to ‘fault system’, introducing a new field in the attribute table (Table 2; Fig. 2a,b).

**Table 2:** Summary of fault trace attributes in AFG. GIS field attributes are also illustrated.

Fault Attributes Table					GIS Field Attributes				
Category	No.	Attribute	Required	Description	Field type	Type name	Length	Precision	Derivation, restricted values
**ID**	1	TraceId	Obligatory	Each fault-trace mapped is given a unique identification number, distinguishing it from other traces. Each trace starts and ends where its geomorphic expression is no longer recognized. Individual traces start/end at natural breaks in geometry.	Decimal (double)	Real	19	0	Unique
**Nomenclature**	2	FaultNo	Obligatory	Fault number is a unique number associated with each fault.	Integer (32 bit)	Integer	7	0	Manually assigned
	3	FaultName	Obligatory	Fault name is based on the first publication (definition) date or, if new, introduced here for first time.	text	String	80	0	Manually assigned
	4	FaultSyst	Optional	Name of the fault system to which the trace belongs, based on structural relationships between faults.	text	String	80	0	Manually assigned
**Geometry**	5	Type	Obligatory	Fault type: normal, reverse, or strike-slip (dextral, sinistral). Minor obliquity is ignored. Ticks on the downthrown side indicate normal faulting, triangles thrust and arrows strike-slip faulting.	text	String	25	0	normal, reverse, dextral strike-slip, sinistral strike-slip
	6	DipDir	Obligatory	Dip direction inferred from downthrown sector. Not registered if strike-slip with no dip component.	text	String	7	0	XXX, where X = N, E, S, or W
	7	Orient	Obligatory	Average fault strike direction, given as a sector of the E hemisphere.	text	String	4	0	XXX, where X = N, E, S, or W
	8	TraceL_m	Obligatory	Length of the individual fault trace (in metres), estimated by QGIS.	Decimal (double)	Real	10	1	Calculated from shapefile geometry
	9	FaultL_km	Obligatory	Length of individaul faults (in kilometres), estimated manually.	Decimal (double)	Real	4	1	Assigned, Measured manually
**Activity**	10	Activity	Obligatory	Interpreted relative activity: “historically active”, “active”, “probably active”, or “uncertain”. Colour-coding: purple, red, green, black (respectively).	text	String	30	0	historically active, active, probably active, uncertain
**Geomorphic Expression**	11	TraceChar	Obligatory (for active faults only)	Describes geomorphic expression of active traces: “sharp”, “moderate”, “rounded”, or “poor”. Colour-coding: red, orange, yellow, blue (respectively).	text	String	10	0	sharp, moderate, rounded or poor
	12	QuatDep	Obligatory (for active faults only)	Indicates whether a fault trace defines a range-front or shows signs of Holocene depositional control. Values: DC (depositional control), or na (not applicable).	text	String	10	0	DC, na
**Supplementary Information**	13	Literature	Obligatory	Reference to relevant publications (citations provided separately).	text	String	254	0	free text
	14	Comm	Optional	General comments on historic ruptures, geomorphic characteristics, etc.	text	String	100	0	free text

### Adopting a definition of “Active faulting” for Greece

#### Approaches to ‘active faulting’

The term “active fault” indicates fault rupture in the relatively recent geological past and also acknowledges the possibility of future rupture (*i.e*.^41,42^). Historically, the term “active fault” has been applied using geological, seismological and historical criteria identifying a fault as a potential source of seismic hazard. Formal definitions of “active faults” contain constraints on temporal activity, which may be dictated by the state of knowledge of geological marker horizons. A commonly used definition for the term “active fault” includes those that have ruptured the ground surface at least once during the last 125,000 years (*i.e*.^43,44^). One advantage of adopting 125,000 years as the boundary between “active” and “inactive” faults is that, in many areas, deposits or surfaces of this age are widespread and easily identifiable (using marginal marine benches or deposits, sea level curves and paleontological, palynological, and/or paleoclimatological data). However, the 125,000 year age limit for active faults may exclude some slow-moving seismogenic faults with long recurrence intervals and destructive potential (e.g. faults in the Basin and Range, USA). Internationally, alternative shorter time-period definitions are also used. In California for example, the legal definition of an “active fault” is one with a proven rupture history during the last 11,000 years (Holocene; https://www.law.cornell.edu/regulations/california/14-CCR-3601). Such refinement is possible where a comprehensive paleoseismological database of fault ruptures has accumulated. This is not the case in Greece.

#### ‘Active faulting’ in Greece

**Fig. 4:**
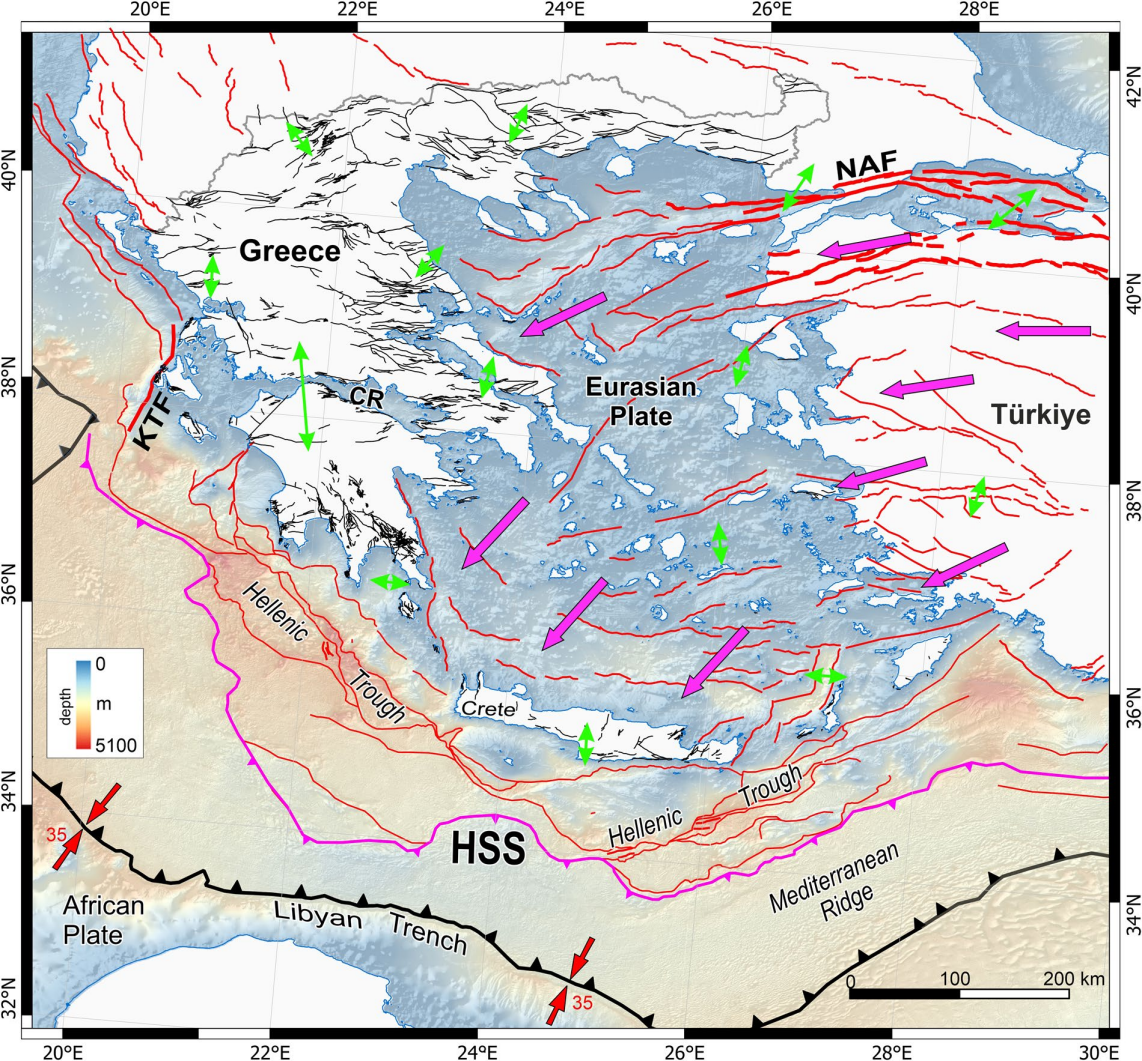
Map summarising the geotectonic setting of Greece and indicating the main tectonic features and kinematics^23,45,47^. Faults in onshore Greece are active traces from this study. Faults offshore Greece and onshore neighbouring countries are summarized from citations^23,45,49,64^. Purple arrows indicate average horizontal GNSS velocities with respect to stable Eurasia while green indicate extension directions^47^. Red arrows represent the orientation of the plate-convergence while numbers the relative rate. HSS = Hellenic Subduction System, KFS = Kefalonia Fault System, NAF = North Anatolian Fault, CR = Corinth Rift.Offshore digital elevation model is from EMODnet (10.12770/ff3aff8a-cff1-44a3-a2c8-1910bf109f85).

Greece is situated on the upper-plate of a convergent plate-boundary along which the African Plate is subducting beneath the Eurasian Plate^45^ (Fig. 4). Arc-shaped plate-convergence marks extensive offshore thrust faulting (i.e. along the Hellenic Trough) and widespread normal faulting landward, towards mainland Greece and the islands^35,37,45,46^. This deformation style is, however, superimposed by two further deformational processes that operate simultaneously, that of the Hellenic slab‐rollback and the westward extrusion of the Anatolian Plate with increasing velocities from East (Anatolia) to south-west (southern Aegean), resulting in extensive normal and oblique-slip faulting throughout continental Greece^47–49^ (Fig. 4).

In an effort to identify chronological markers and establish relative chronology of faulting on the landscape of Greece, some workers have used rupture younger than the last glacial maximum (LGM = ~18 thousand years) in separating “faults with post-glacial activity” from older faults. This concept, originally proposed in Greece by Armijo *et al*.^46^, reflects the conceptual initiation of a period of landscape stability (post-LGM) following elevated climate-induced landscape instability during the last glacial period, has been tested and used by many studies in Greece and the circa-Mediterranean subsequently (*i.e*.^35,37,50–56^). In parts of Greece, such as Crete and the Peloponnese, these “post-glacial traces” in limestone substrates are represented by clearly mappable elongate ribbons of bedrock exposure, visible as pale, bare or poorly vegetated scarps traversing the landscape.

As a mountainous country with varying basement lithologies there are, however, many parts of the Greek landscape that lack youthful chronological markers. Much of the country has been terrestrial for at least the last 5 million years and thick sequences (>2 km) of alluvial and/or terrestrial deposits are present in many places. Fault-traces restricted to mountainous areas are commonly preserved only as lineaments in the landscape (i.e. topographic fault-scarps) and/or as fault damage zones in bedrock. The longevity of a fault-scarp or fault-line scarp in bedrock depends on the resistance to erosion of the rock itself, as well as the local erosional regime^28^. In some lithologies and erosional circumstances, fault traces may last for hundreds of thousands of years, while in others they will disappear within decades to hundreds of years. Furthermore, the expression of an old inactive fault in the landscape may be exaggerated by differential erosion where contrasting lithologies are present on either side. To better understand the persistence of fault scarps in varying materials and our ability to detect them using available data, we assessed the geomorphic expression of the fault traces associated with each historical surface-rupturing earthquake in Greece. In many cases, we found that surface ruptures recorded in the literature for large earthquakes in the last 2500 years are difficult to identify in the available DEMs and satellite imagery.

**Fig. 5:**
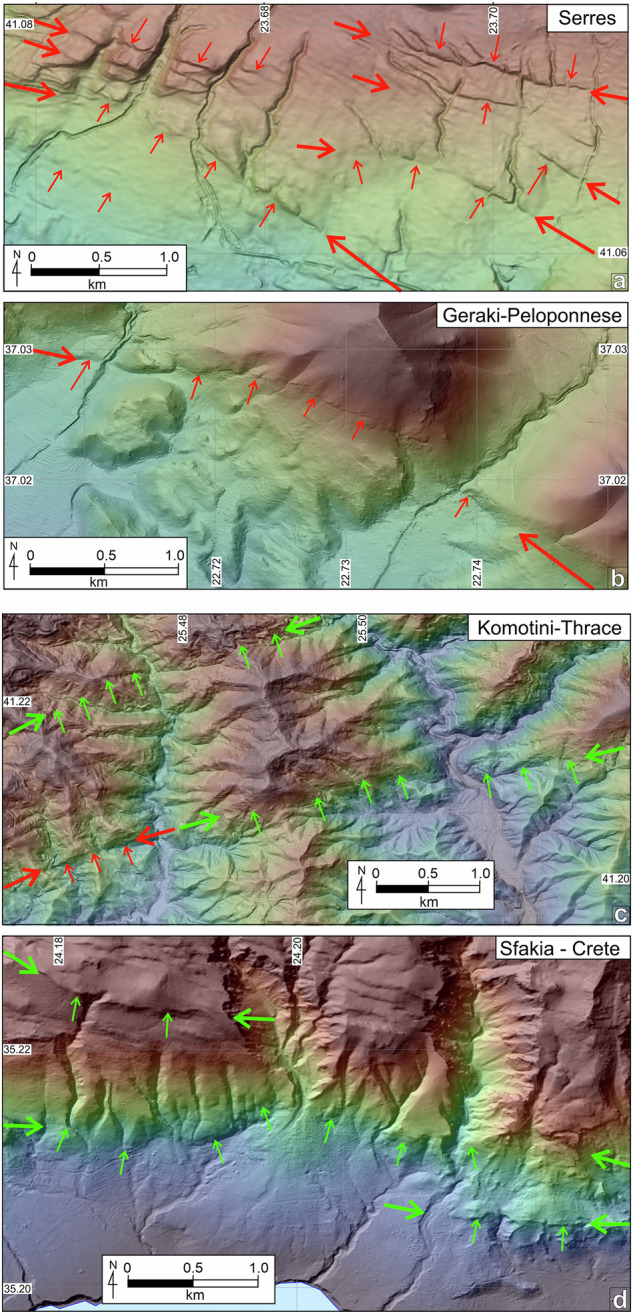
Examples of “*active*” and “probably active” fault traces in the landscape. (**a**) Multiple sharp active traces of the Serres Fault crossing a Quaternary fan in the Serres area (2.5 km NE from Chryso). Note that fault traces displace fans, that retain their depositional morphology. (**b**) The West Parnon Fault displaces youthful alluvial deposits where it crosses two small streams 4 km NE of Geraki in the central Peloponnese. Active faults in (a) and (b) are marked close to the edge of the figure with heavy red arrows; traces are indicated using lighter arrows at right angles along their length. (**c**) The Kavala-Xanthi-Komotini Fault System in Thrace (northeast Greece), includes active and probably active fault traces. The western trace in this image, about 14 km northeast of Komotini, has a sharp geomorphic expression and is clearly active; its northeast extension lacks this character, nevertheless, facetted spurs, an abrupt change in land elevation and river entrenchment strongly indicate the presence of a fault. Another trace of similar strike about 1.8 km to the north, is also attributed as probably active. (**d**) Traces of the Sfakia-Sella Fault System in southwest Crete (Sfakia region) features prominent triangular facets and a clear topographic signature without, however, a clear continuous active trace with identifiable postglacial scarps. The coordinate system used is WGS 84, EPSG: 4326.

In the absence of comprehensive and accurate, nation-wide, youthful geochronological markers a philosophical decision was required on how inclusive the database should be. The decision influences whether all active or potentially active faults are included within the database, or merely the largest and most obvious active faults. To avoid the possibility of omitting significant seismic sources, we have chosen to be inclusive, and extend the time-window of ‘active faulting’ to include the Quaternary (i.e., since 2.58 Ma). This definition was adopted because faulted Quaternary *alluvial fans,* that retain their original fan morphology, and *lowland basins* provide widespread chronostratigraphic markers in Greece (note that although these a*lluvial fans* are considered Quaternary in age, most likely they are considerably younger). In AFG, fault-traces displacing these fans are designated as ‘active’ (Fig. 5a). Fans adjacent to *lowland basins* commonly exhibit gently undulating surface morphology and likely represent areas of late Quaternary deposition (probably < 70,000 years, the start of the last glacial period of landscape modification). Where these surfaces are displaced by faults, there is little doubt that they should be regarded as ‘active’, even using the commonly accepted criterion of 125,000 years*. Lowland basins* are normally sites of deposition, so existing surfaces are necessarily young (in many cases they are inferred to be Holocene in age). Fault traces preserved across these surfaces are considered “active”, while many traces buried by ongoing deposition may also be “active”. Our geomorphological mapping is unable to locate these concealed active faults, although in some cases their existence can be inferred by along-strike projection of fault traces into the basin. Further, all traces characterized as ‘sharp’ (see following section) are potential indicators for late-Pleistocene and/or Holocene activity and should also be considered as “active”.

Thus, within the AFG active faults are those that have ruptured the surface during the Quaternary, leaving remnants of geomorphic expression (note that some may not qualify as “active” using strict application of definitions described above). These faults warrant further investigation (e.g., constrain the timing of their last rupture and/or their paleoearthquake history, earthquake recurrence interval, earthquake magnitude, etc) to better understand the hazard they represent. For the purposes of seismic hazard assessment, in the interests of inclusivity, named faults that have active fault traces should be regarded as active unless otherwise proven. Of the 892 faults in the database, 54% have active traces and thus should be regarded as active as defined within this database; the other 46% may be optionally treated differently within the seismic hazard assessment, or excluded from the assessment altogether.

### A classification of fault activity in AFG

Assigning fault activity involved checking for scarps across Quaternary deposits, or for facets on bedrock spurs, where lineaments are present at the margin of basinal deposits. Here, where either a scarp in Quaternary sediments or fault facets were identified, the fault was described as “*active*”. Where younger materials are not displaced, a trace may be defined as “active” when: a) it is located directly along-strike from a demonstrably active trace; b) there is a clear structural connection with demonstrably active traces; and/or c) when there is a clearly ‘sharp’ trace (i.e., earthquake fault-scarp) identified somewhere along its length (Fig. 5a). Fault traces that do not displace young sediments, do not meet the above criteria or whose extent is limited to land underlain by bedrock, are characterised as “*probably active*” (Fig. 5b). Future investigation of individual fault traces designated as “probably active” in our dataset may change their attribution to “*active*” (or vice versa). Fault traces that we attribute as “*uncertain*” represent features that do not meet any of the above criteria and, further, there is uncertainty whether they represent stratigraphy and not faulting (i.e., represent an erosional expression of contrasting lithologies) or whether they are relict older faults or folds that are no longer active. While there is no intention to include inactive faults in the AFG, it remains possible that the dataset contains some of these older faults.

### A geomorphic classification of active fault traces

**Fig. 6:**
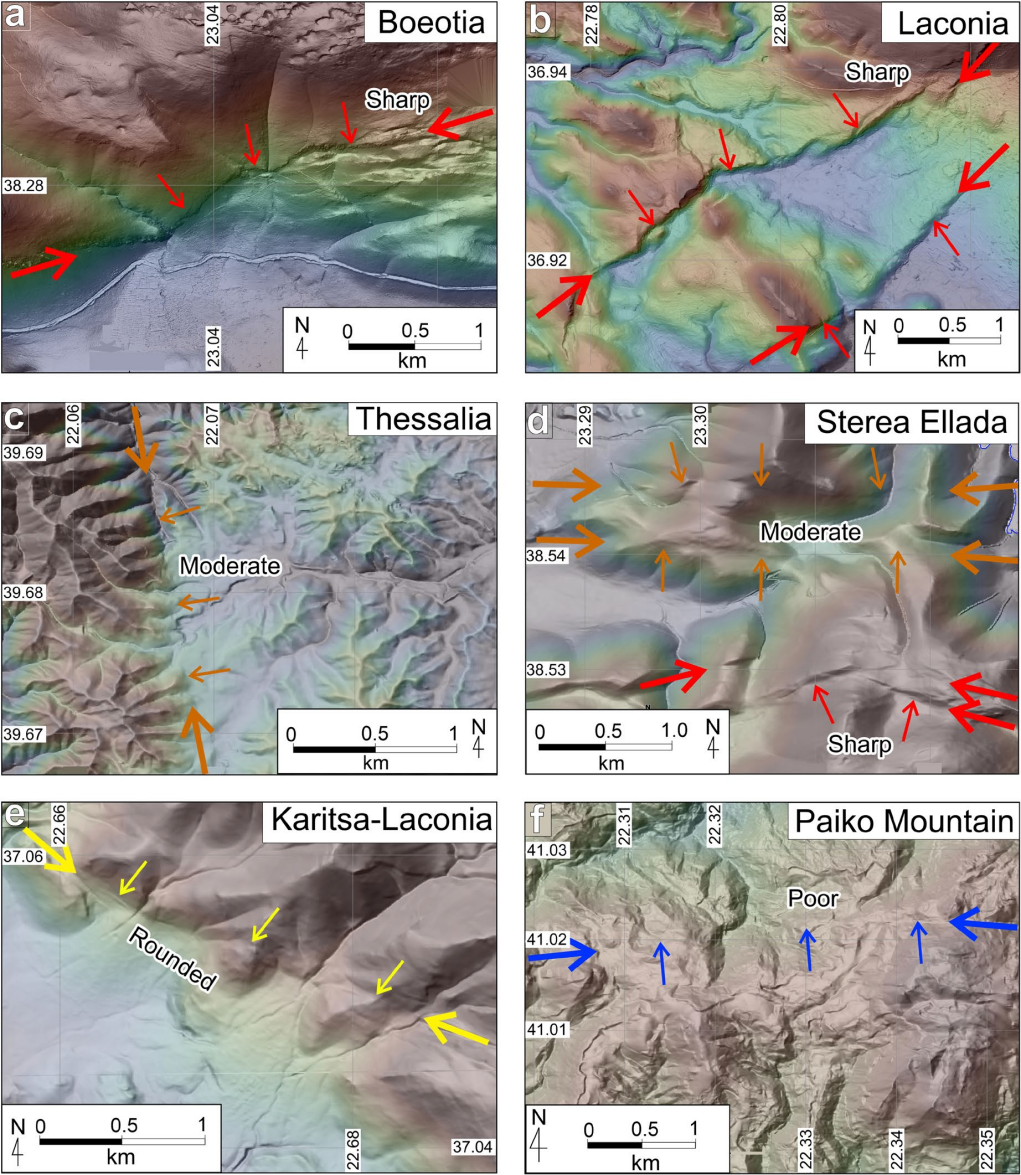
Examples of varying geomorphic expressions of active faults. Maps illustrate typical examples of “*sharp*” (**a,b**), “*moderate*” (**c,d**), “*rounded*” (**e**) and “*poor*” (**f**) traces of active faults in AFG. Heavy arrows along the strike of traces identify the fault, while lighter arrows at right angles mark the location of the fault trace. Arrows are colour-coded according to the geomorphic expression of the trace (red = sharp, orange = moderate, yellow = rounded, blue = poor). (**a**) Traces of the Neochori-Leontari Fault about 5.5 km southwest of Neochori (Boeotia Prefecture) with a sharp geomorphic expression on the landscape; (**b**) Active traces of the Apidea Fault System in Lakonia (Eastern Peloponnese) sharply displace Quaternary fans and streams; (**c**) trace of the Farkadona-Kalyvia Fault System approx. 1.6 km southwest of Megalo Eleftherochori in Thessalia traverse dissected hill country, exhibiting facetted spurs but a rounded geomorphic expression; (**d**) Traces of the active Lokris and northern Orchomenos fault systems in central Greece (Sterea Ellada), displacing ridge-crests that have “*moderate*” geomorphic expressions (orange arrows), while the traces of the southern Orchomenos Fault are markedly clearer and are characterised as “*sharp*” (red arrows); (**e**) The active trace with “rounded” geomorphic expression of the Karitsa Fault System in south Peloponnese (Laconia - 5.4 km northeast of Agioi Anargyri). The fault traces displace ridge systems and active fans, and bound entrenched streams; (**f**) The “*poor*” geomorphic expression of the Belles Fault System in Paiko Mountain Range in Macedonia: although its traces appear to clearly displace a series of ridges, its landscape expression is limited and difficult to locate between displaced features. Coordinate system: WGS 84, EPSG: 4326.

To assign confidence level on our fault trace mapping, we have introduced a qualitative geomorphic trace descriptor. This geomorphic classification was only undertaken for faults attributed “*active*” or “*historically active*” (the latter where discernible). Where historically active traces are not resolvable, they are attributed as “not applicable” (na). We have assigned to active and historically active faults the primarily qualitative geomorphic descriptor of “sharp”, “moderate”, “rounded” or “poor” to reflect geomorphic expression in the landscape. The “sharpness” of expression of fault traces in natural situations is a continuum and the qualitative descriptors are necessarily imprecise, with overlap between these terms. Examples of each geomorphic descriptor are illustrated in Fig. 6. Where a trace is “sharp” anywhere along its length, the entire trace is considered “sharp”. The same rule is applied to other options in this field (i.e., in the progressive sequence from “sharp”, “moderate”, “rounded” to “poor”). In terms of its use as an indicator for accuracy of line location, we estimate that “sharp” and “moderate” traces are located within 100 metres of the fault itself. “Rounded” and “poor” traces have a less certain location, which we estimate to range from 100 to 1000 meters. Further, some inference for timing of rupture may be gained from the geomorphic expression of fault traces. The classifications provided may be used as qualitative estimates for the timing of the last rupture. Faults attributed as “sharp” may be interpreted as having ruptured within the Holocene, for example, while those with “rounded” attribution probably last ruptured substantially earlier (e.g., 100 kyr to 1 Myr ago). Although clearly, a “sharp” scarp will remain longer in the landscape on competent erosion-resistant rocks than in soft sediments, a qualitative use of this geomorphic descriptor field may help identify sites suitable for future paleoseismological studies. In summary, these descriptors can be viewed as indicators of the ‘confidence level’ with which each trace is represented on the landscape.

To complement the geomorphic analysis, we have also assessed whether fault traces exert control on local sediment deposition (depositional control – DC) (Tables 1, 2; Fig. 1). Where a trace defines a range-front between hill/mountain and Quaternary depocenters or shows indications of Holocene depositional control (interpreted as likely to be an association between fault development and deposition), it is attributed as DC (Fig. 1). As we have done for the sharpness descriptor, any fault-trace with evidence indicating DC anywhere along its length, is attributed DC in its entirety. On the other hand, where there is either no recent deposition along the length of the fault trace or no unequivocal indication of fault-controlled deposition the trace is characterised as ‘na’ = ‘not applicable’ (Table 2).

#### A geomorphological caution - Landscape modification

The long human occupation of Greece (>5000 yrs) has implications for active fault preservation. Significant historic (<1500 years) and pre-historic (1500 to 5000 years BP) anthropic landscape modification is recorded in many parts of Greece^57,58^. Such modification has changed the original surface expression of faults, particularly of lowland faults (in intensively cultivated areas), across much of the country. Because active fault ruptures are commonly small in displacement, they are particularly prone to anthropic modification. The impacts of anthropic modification include soil development and soil loss leading to degradation of fault scarps on flat or gently sloping landscapes, terrace-building and other constructions that disguise the presence of faults, and consequent blurring or obliteration of geomorphology useful in defining faults as ‘active’. Local studies, mostly for the purposes of archaeology^57,58^, provide a local basis for understanding the impact and chronology for potential soil development and thus fault scarp modification.

## Data Records

The dataset is available at GFZ Data Services^59^ in the format of an ESRI shapefile, Google Earth kmz and Excel formats. The associated reference list AFG is presented in the Supplementary Information of this article in Word format. Further, the AFG may be viewed and queried on an ArcGIS webserver that is publicly available through the GFZ Data Services^59^.

### Fault attributes

All fault traces were mapped using 2 m and 5 m DEMs and derivative attributes were generated as described above. The database includes a total of 3815 trace records, assigned to 892 individual faults and 236 fault systems (Table 1), with each individual trace containing 14 attributes (Table 2; the GIS field attributes are also included). The AFG attribute table, assigns obligatory and optional fields for each trace, including descriptors of ID, name, geometry, type, activity, geomorphic expression, and reference to previous work with associated comments. The following fields are present in the ESRI shapefile, and below are grouped to illustrate the overall structure of the attribution (Fig. 2a; Table 2).

1. ***TraceId:*** Obligatory field. Each fault-trace mapped is given a unique identification number (Unique ID), distinguishing it from other traces; each starts and ends close to where its geomorphic expression can no longer be recognised; individual traces start and end at or near a natural break in their geometry.
  **Name**
2. ***FaultNo:*** Obligatory field. Fault number provides a unique ID for each interpreted fault (that, in many cases, comprises multiple fault traces; see Fig. 2b).
3. ***FaultName:*** Obligatory field. Fault name, where present, determined by first publication (definition) date. Where previously unnamed, a name is selected from a nearby village or town.
4. ***Fault_Syst:*** Optional field. Fault system name; fault system for grouped individual faults, allocated based on perceived structural relationships between traces and faults.
  **Geometry**
5. ***Type:*** Obligatory field. Fault type includes normal, reverse or strike-slip (dextral or sinistral); minor components of obliquity are ignored.
6. ***DipDir:*** Obligatory field. Dip direction of the fault-trace plane, interpreted from the defined downthrown sector. The only case where no dip direction may be registered is where a fault is considered strike-slip, with no significant dip slip component.
7. ***Orient:*** Obligatory field. Generalised average fault-trace strike expressed as a sector of the E hemisphere.
8. ***TraceL_m:*** Obligatory field. Individual fault trace length estimates (in metres) calculated automatically by QGIS software.
9. ***FaultL_km:*** Obligatory field. Individual fault length estimates (in kilometres), measured manually for each fault.
  **Assessment of activity**
10. ***Activity:*** Obligatory field. Interpretation of relative activity based on historic and geomorphic characteristics; field options include “*historically active*”, “*active*”, “*probably active*” or “*uncertain*”. Colour-coding: purple, red, green, black (respectively).
  **Geomorphic expression**
11. ***TraceChar:*** Obligatory only for “active” faults. Qualitative descriptor of the geomorphic expression of active fault traces; where applied, options are either “*sharp*”, “*moderate*”, “*rounded*” or “*poor*”. Colour-coding: red, orange, yellow, blue (respectively).
12. ***QuatDep:*** Obligatory field only for “active” faults. Records fault traces defining a range-front between hill/mountains and Quaternary depocentres or shows indications of Holocene depositional control; depositional control – DC; na = not applicable (either due to no Holocene deposition along the length of the trace or no unequivocal indication of fault control of deposition). Any fault with evidence for DC anywhere along its length is attributed DC in its entirety.
  **Supplementary Information**
13. ***Literature:*** Obligatory field. Reference to relevant publications (full citations are listed in an accompanying Word file).
14. ***Comm:*** Optional field. General comments noting historic ruptures, or specific geomorphic or other characteristics of the fault-trace.

### Fault distributions and trends

**Fig. 7:**
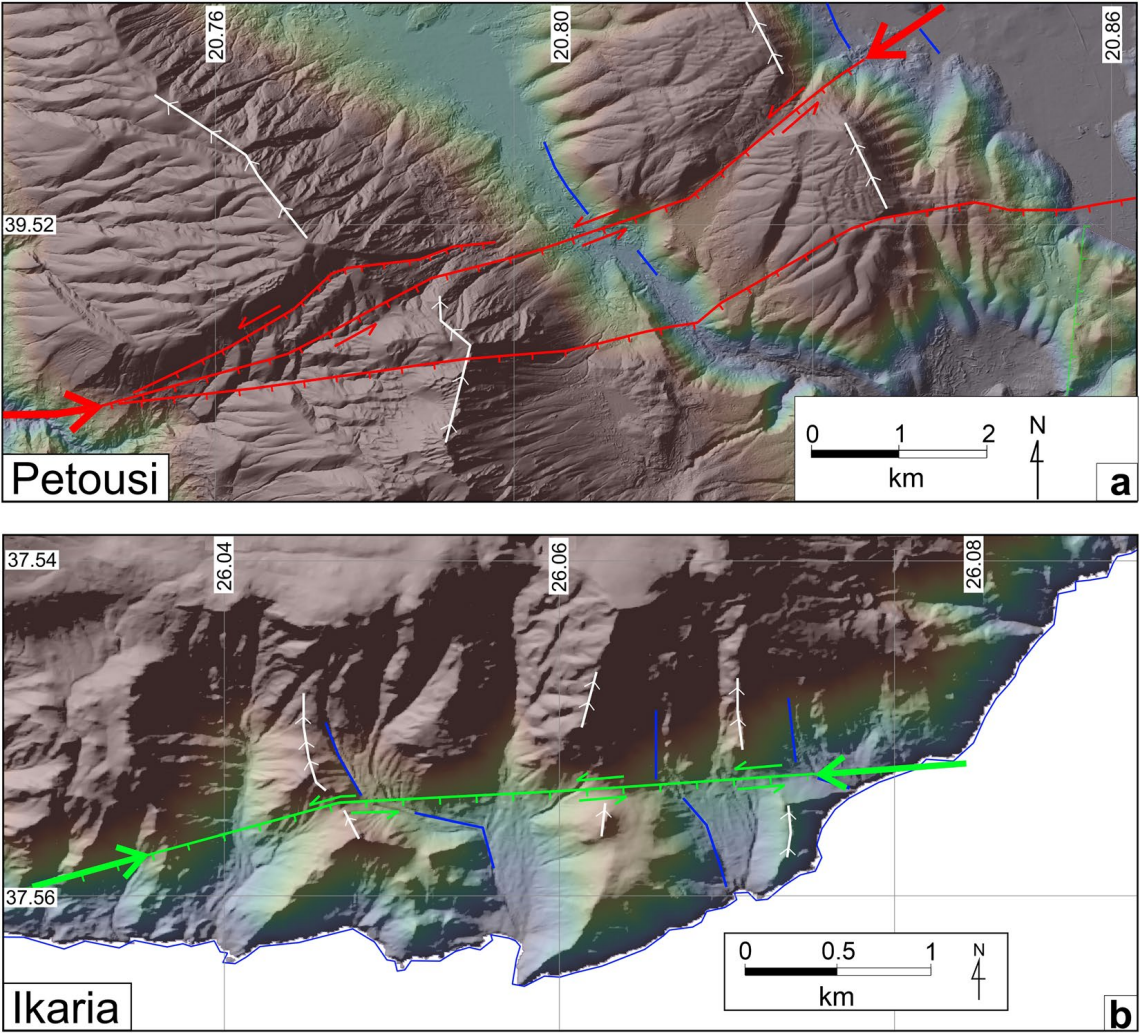
Example of possible strike-slip displacements. (**a**) An apparent sinistral displacement of ridge-crests (white lines with ticks) and valley margins (blue lines) is present across active traces of the Pertouli-Souli Fault System between the villages of Tseritsana and Episkopiko (Kalamas Prefecture). It is likely that these displaced landscape features result from an earlier phase of fault activity (i.e. there is no clear lateral displacement of younger landscape features). (**b**) On southwestern Ikaria Island, between Karkinagri and Trapalo, a probably active trace displaces left-laterally ridge-crests and stream-beds (indicated by white and blue lines, respectively). Active faults in (**a,b**) are marked close to the edge of the figure with heavy red arrows. The coordinate system used is WGS 84, EPSG: 4326.

**Fig. 8:**
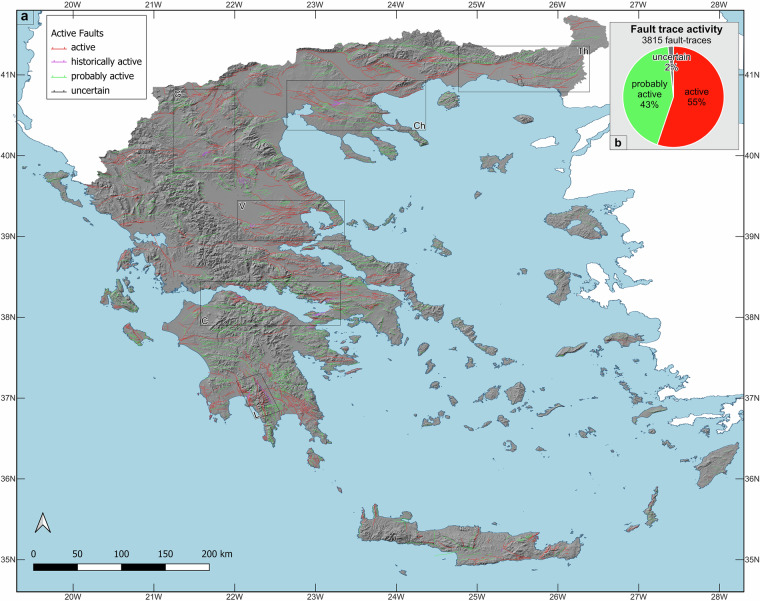
**(a) **The AFG database. Red lines illustrate “*active*” fault traces, green lines “*probably active*” traces, black lines denote “*uncertain*” faults while magenta lines indicate “*historic ruptures*” (for a complete list see Table S1 in the SI). (**b**) Chart illustrating the contribution (%) of each type of fault activity in AFG. The hillshade on the basemap was created using 30 m Shuttle Radar Topography Mission (SRTM GL1) Global elevation data available from Open Topography (https://portal.opentopography.org). Six black boxes indicate the regions sampled for the analysis presented in Fig. 11a: G = Grevena, Th = Thrace, V = Volos, Ch = Chalkidiki, C = Corinth, L = Laconia. For high-resolution version of this figure see https://datapub.gfz.de/download/10.5880.FIDGEO.2025.047-VEnuis/.

**Fig. 9:**
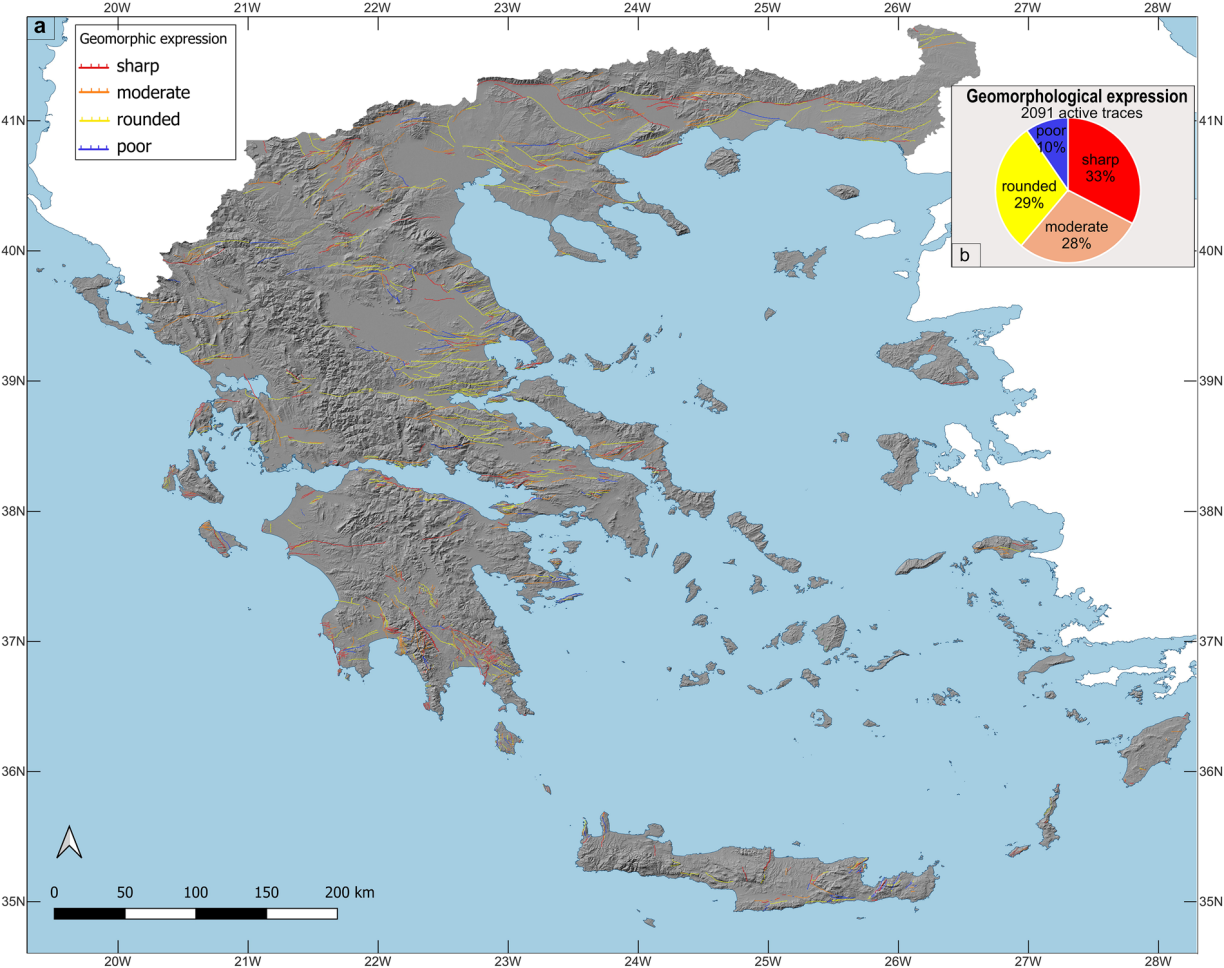
**(a)** Geomorphic expressions in AFG. Figure illustrating active fault traces characterized by their geomorphic expression as sharp (red), moderate (orange), rounded (orange) and poor (blue). (b) Chart illustrating the trace character contribution (%) in the distribution of active faults. The hillshade on the basemap was created using 30 m Shuttle Radar Topography Mission (SRTM GL1) Global elevation data available from Open Topography (https://portal.opentopography.org). For high-resolution version of this figure see https://datapub.gfz.de/download/10.5880.FIDGEO.2025.047-VEnuis/.

The vast majority of the AFG traces (>99%) are normal faults, with only two trace entries of reverse faults and 20 of strike-slip faults, which make up one reverse and six strike-slip faults, respectively (Table 1 and Supplementary Table S1). Although strike-slip displacements are difficult to detect in Greece’s landscape, in Fig. 7 we present clear evidence for horizontal displacements on two individual faults: (1) The Petousi Fault System in Epirus (western Greece), where sinistral displacement of ridge-crests and valley margins are recorded (Figs. 7a) and (2) The Karkinagri Fault at the south Ikaria Island that exhibits consistently sinistral offset of spurs and drainage channels (Fig. 7b). In contrast, the large right-lateral strike-slip faults of Ilia-Achaia (Peloponnese) and Strymon (Macedonia) could not be identified at the ground surface, and their location was approximately positioned using the aftershock sequence of the 2008 M6.4 earthquake^10,29^ and geodetic observations^60^. Only two trace-records, part of the same fault on Zakynthos, are attributed to thrust mechanisms (Supplementary Table S1); a correlative fault ~10 km to the south, is interpreted as a thrust in offshore seismic-reflection profiles^61^. Figures 8, 9 illustrate the AFG queried as per fault activity and geomorphic expression, respectively. In Fig. 8 “*active*” fault traces appear red, “*probably active*” fault traces green, and “*uncertain*” fault traces are illustrated black, while “*historic ruptures*” are shown in purple. Here, “*active*” and “*probably active*” faults dominate, with the “*active*” faults being slightly more abundant than the “*probably active*” (55% vs. 43%). Figure 9 suggests that “sharp” (red), “moderate” (orange) and “rounded” (yellow) traces appear to be approximately equally distributed throughout the active faults of Greece (Fig. 9, Table 1), with the location of the sharp traces potentially indicating sites for future paleoseismic trench excavations. Of the discernable historical fault ruptures, half are characterised as sharp, the other half as rounded (Supplementary Table S1).

## Technical Validation

**Fig. 10:**
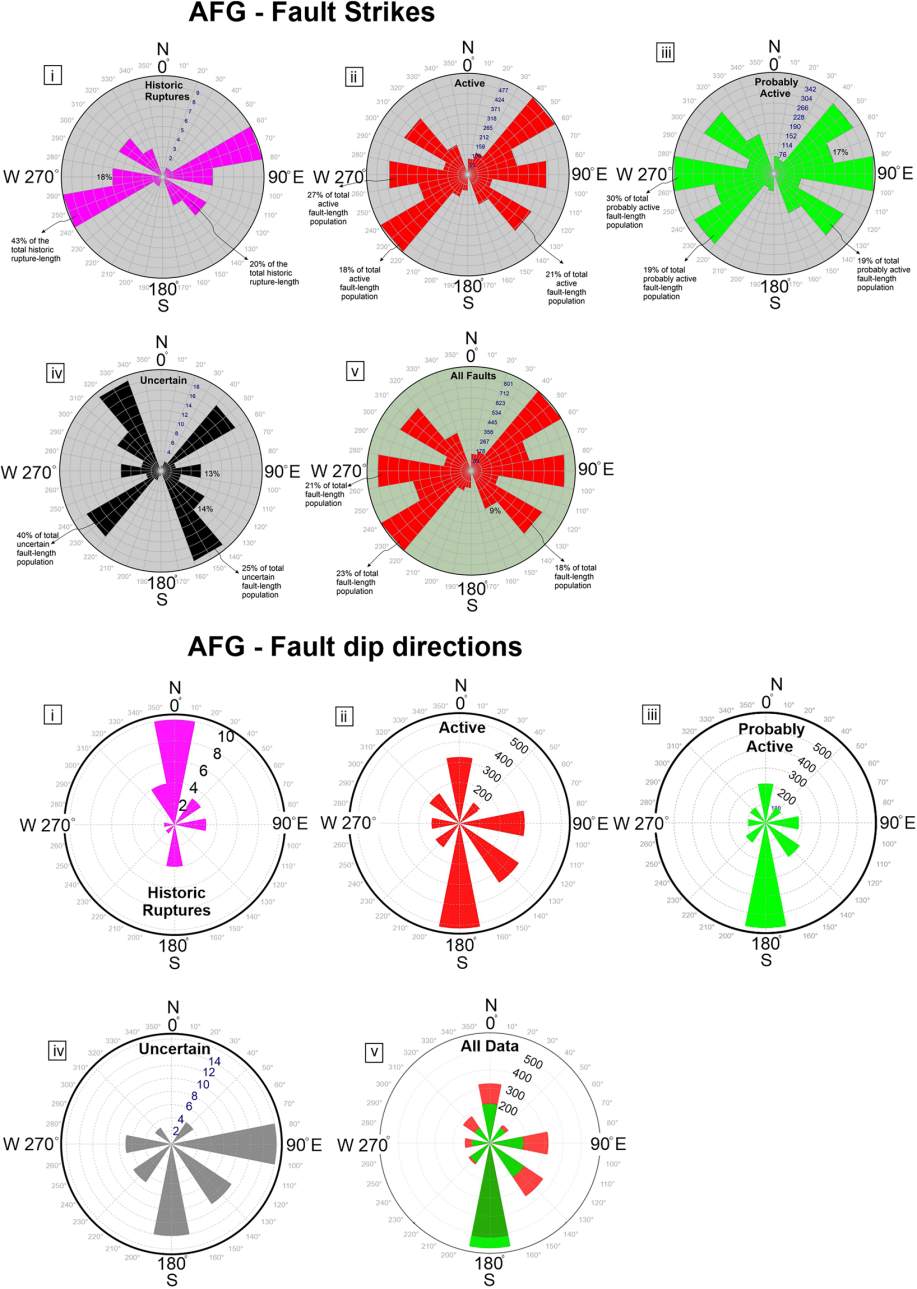
Principal fault geometries. (**a**) Rose diagrams illustrating the prevailing fault-trace strikes in AFG as per different fault activity (i-iv) and collectively for the entire dataset (v). (**b**) Rose diagrams illustrating the prevailing fault-dip directions in AFG as per different fault activity (i-iv) and collectively for the entire dataset (v).

Normal faulting in Greece largely reflects active multi-directional extension, with a predominance in NW-SE and N-S extension (Figs. 4, 8–10). Specifically, the dominant strike of the “*active*” fault traces is NE-SW, representing however only 18% of the total active fault-trace length population (because these fault traces are short), whereas for the “*probably active*” fault traces the dominant strike is E-W, amounting to 30% of the total fault lengths (Fig. 10a). The dominant strike of historic ruptures is also NE-SW (rotated slightly more eastward than the “*active*” fault traces), accounting for about 45% of the historic rupture lengths. Fault traces listed with “*uncertain*” activity represent only 2% of the database and are found largely on Kefalonia, Peloponnese and some islands (Kythira, Rhodes, Amorgos and Chios). “Uncertain” faults are typically shorter than most “active” or “probably active” traces, with 14 of these traces showing some indication of depositional control (i.e. they may be active). The dominant strike of “uncertain” faults is NW-SE (i.e. approx. perpendicular to the dominant trend of the “active” and “probably active” faults), representing about 25% of the “uncertain” trace-length population. Fault-dip directions for normal fault traces in the AFG mainly dip to the south, while historic ruptures form an outlier (compared to active faults) as they mostly dip to the north (although responding to the same N-S extensional field as the active faults) (Fig. 10b).

**Fig. 11:**
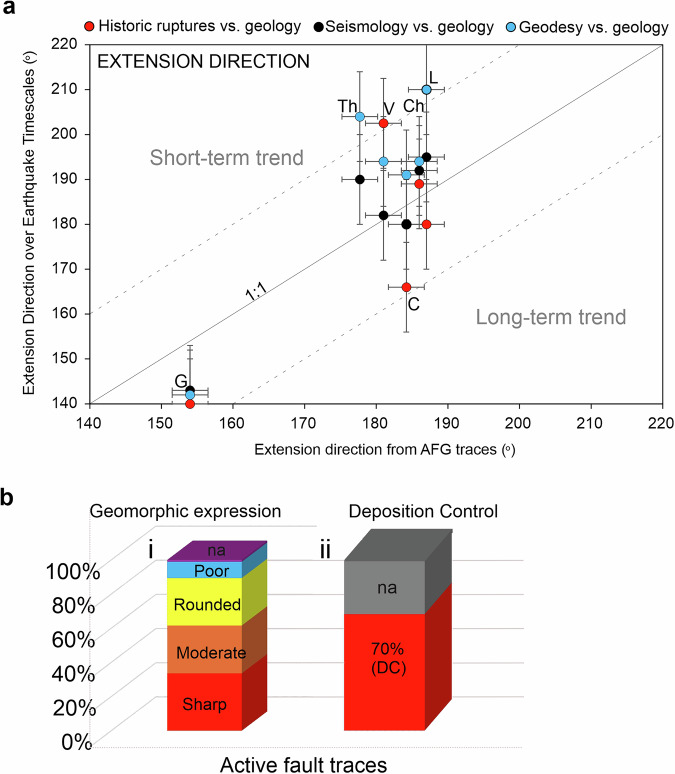
Validation of fault geometries and fault-trace character. (**a**) Mean extension direction of the AFG traces included in each of the six regions sampled (see Fig. 8 for location), plotted against the mean extension direction (for the same regions) of historic ruptures (red), geodetic measurements (blue) and inversion of seismological data (black). Dashed lines represent ~20° uncertainty. G = Grevena, Th = Thrace, V = Volos, Ch = Chalkidiki, C = Corinth, L = Laconia. (**b**) Histograms illustrating the percentage of sharp and moderate traces (62%) in the active fault AFG population (i) and the percentage (70%) of ‘deposition control’ (DC) for the entire AFG (ii).

To test whether the geometry and kinematics of the active fault traces included in AFG agree with fault attributes that derive from different methodologies, we compare the mean extension direction from six regions in AFG (Corinth, Chalkidiki, Laconia, Volos and Thrace; Fig. 8), with the extension direction that derives in each region from independent geodetic and seismological datasets and historical ruptures (Fig. 11a). Specifically, assuming pure normal faulting, we have calculated, using the trend of the mapped AFG fault traces, the extension direction in each selected region and compared it with extension directions that derive from geodetic measurements^47^, inversion of earthquake moment tensors^48^ and that of historic ruptures (this study). Comparison shows that in all six cases there is a good agreement (within ±20°) between the mean extension direction in AFG database and that calculated over significantly shorter timescales (i.e. ‘earthquake timescales’), here represented by historic ruptures (red datapoints), geodetic (blue datapoints) and seismological data (black datapoints) (Fig. 11a). The vertical separation of each datapoint from the ‘1:1’line denotes the uncertainty between the mean trend of the AFG traces and each sampled ‘short-term’ dataset whereas the vertical separation of the datapoints in each region reflects uncertainties arising from using various datasets (note that in all six cases deviations are ≤30° and mostly ≤20°). This is also reinforced by the mean trend of the historical ruptures which is comparable to the dominant trend of the active fault traces. These comparisons provide a first-order validation of our fault mapping and cannot work in regions of Greece where normal faulting is multi-directional (i.e. southeast Peloponnese; Fig. 8).

**Fig. 12:**
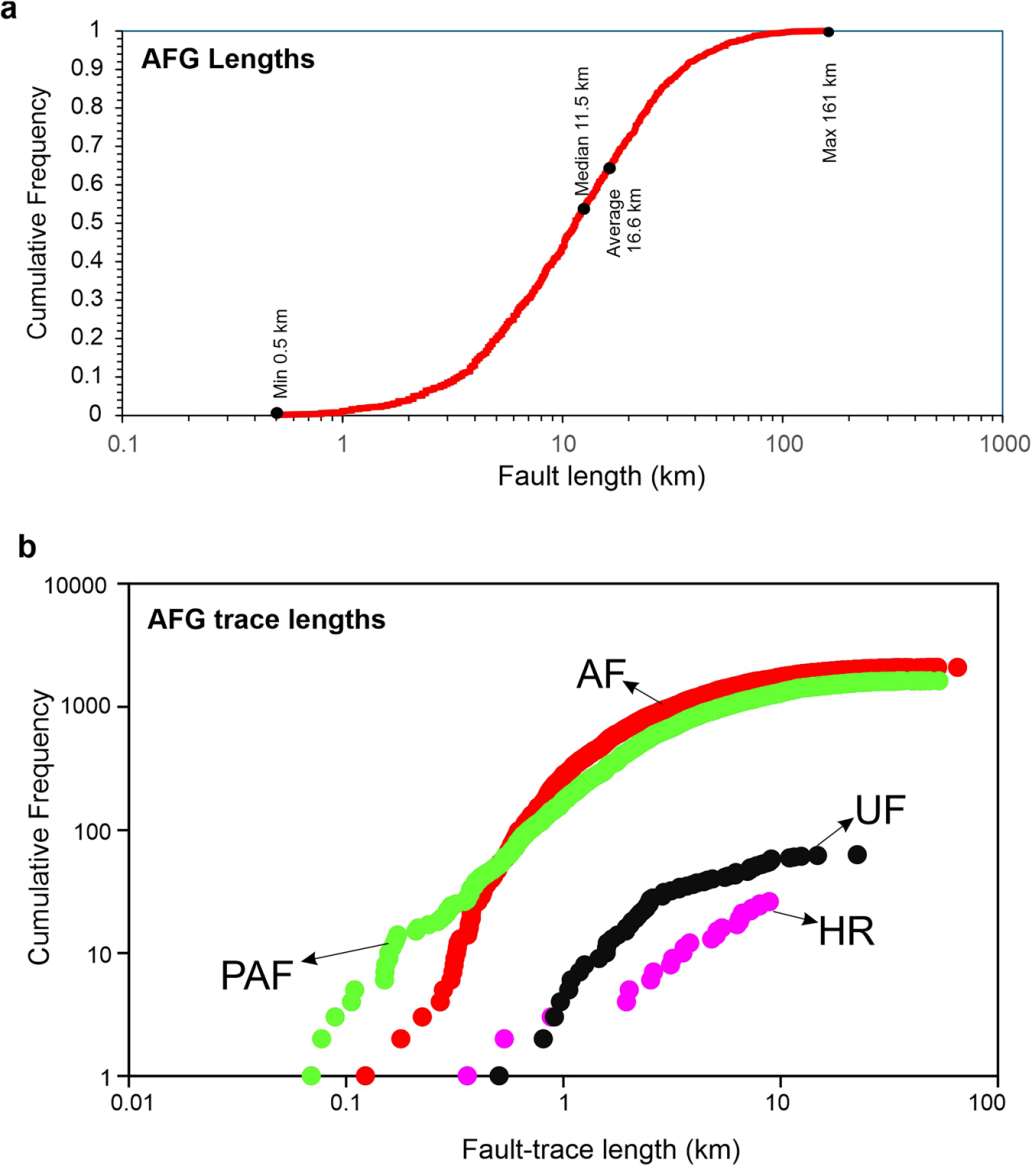
Fault lengths and fault trace lengths in AFG. (**a**) Normalised cumulative distribution of AFG fault lengths. Min-max, median and average values are indicated. (**b**) Log-log plot of cumulative frequency of fault-trace lengths colour-coded as per Fig. 10. AF = “*Active Faults*”, PAF = “*Probably Active Faults*”, UF = “*Uncertain Faults*” and HR = “*Historic Ruptures*”.

To further validate the mapped AFG traces we have tested the geomorphology of the “*active*” fault traces (red in Fig. 8) against a series of qualitative geomorphic attributes introduced in Table 2. Our fault mapping may be rationalised if most ‘*active*’ faults exhibit “*sharp*” or “*moderately sharp*” traces with “*deposition controlled*” activity (DC). Indeed, analysis suggests (Fig. 11b) that the majority (62%) of the mapped AFG traces are well imprinted on the landscape while 71% of them are characterised by deposition control (DC). Fault lengths in AFG range from 500 m to >150 km, with the average fault length at 16.6 km and the majority (80%) shorter than 25 km (Fig. 12a). The “active” and “probably active” fault-trace length populations indicate power-law scaling, in agreement with other global and local fault compilations^62,63^, while the historic ruptures are truncated due to limited sampling resolution (Fig. 12b). Further, we have georeferenced and compared our traces with all previously published faults. Comparison reveals a good agreement between the two independently derived fault datasets and, in most cases, AFG improves the location of previously published faults (often by several km’s) because its traces are tailored to specific geomorphological markers (Figs. 5–7).

In summary, the AFG is a database designed to provide the best available data for those seeking to work further on faults in Greece. While only a subset of traces (c. 45%) has been field-checked, the database is based on geomorphological data that can be independently checked by potential users. We have confidence that the geomorphic features we have used to identify fault traces (e.g. displaced surfaces, linear slope changes, offset ridges and streams) are reliable indicators. Historical ruptures provide validation for some of the faults but future large-magnitude earthquakes in onshore Greece will provide the ultimate test for some of our proposed fault traces and their attribution.

## Usage Notes

Because AFG aims to be a long-term community-based project, it can be viewed and queried through an online web-map server^59^, from where data and metadata may be extracted in the form of shapefiles or kmz files. The AFG metadata (including a comprehensive reference list) are attached to the shapefile and also stored at the GFZ Data Services website^59^ (10.5880/fidgeo.2025.047). Restrictions apply to the availability of the Digital Elevation Models (DEMs) used to map the fault traces. These data belong to the Greek Cadastre Agency and may be accessed upon request to the agency.

## Supplementary information

Reference list S1_REFS

Supplementary Table S1

## Data Availability

The AFG dataset is available at the GFZ Data Services website^59^ (10.5880/fidgeo.2025.047). Note that the AFG may be viewed and queried on an ArcGIS webserver that is also publicly through the GFZ Data Services website^59^.

## Code availability

For producing the fault maps in Figs. 3–9 we used the open-source software QGIS (2020): Version 3.16 [https://www.qgis.org/]. Hillshades presented in Figs. 5–7 are from DEMs provided by the Greek Cadastre and are available upon request. The hillshade on the basemap in Figs. 8, 9 was created using 30 m Shuttle Radar Topography Mission (SRTM GL1) Global elevation data, available from Open Topography (https://portal.opentopography.org). The dataset is freely available at 10.5880/fidgeo.2025.047 under a Creative Commons Licence in ESRI shapefile, kml and Excel formats.
