# Supplementary material for: AFG - Active Faults Greece: a comprehensive geomorphology-based 1:25,000 fault database
Source: Sci Data. 2025 Nov 21;12:1853. doi: 10.1038/s41597-025-06283-z (PMC12639124; doi:10.1038/s41597-025-06283-z)
Supplement: Supplementary file 1 — Reference list S1_REFS [file 41597_2025_6283_MOESM1_ESM.docx]

# AFG References

1. Ambraseys, N. N. 2001. Reassessment of earthquakes, 1900-1999, in the Eastern Mediterranean and the Middle East. *Geophysical Journal International,* **145,** 471-485.
2. Armijo, R., Lyon-Caen, H., & Papanastassiou, D. 1991. A possible normal-fault rupture for the 464 BC Sparta earthquake. *Letters to Nature,* **351**.
3. Basili, R., Kastelic, V., Petricca, P., Tarabusi, G., Tiberti, M., & Valensise, G. 20136. The European Database of Seismogenic Faults (http://diss.rm.ingv.it/share-edsf/). On-line database;10.6092/INGV.IT-SHARE-EDSF.
4. Begg, J., Mouslopoulou, V., Heron, D., & Nicol, A. 2025. Active Faults Greece (AFG): a comprehensive geomorphology-based 1:25,000 fault database. *Scientific Data (in review)*.
5. Benedetti, L., Finkel, R., Papanastassiou, D., King, G., Armijo, R., Ryerson, F., Farber, D., & Flerit, F. 2002. Post-glacial slip history of the Sparta Fault (Greece) determined by ^36^Cl cosmogenic dating: Evidence for non-periodic earthquakes. *Geophysical Research Letters,* **29**, 8, 10.1029/2001GLO14510.
6. Benedetti, L., Finkel, R., King, G., Armijo, R., Papanastassiou, D., Ryerson, F. J., Flerit, F., Farber, D., & Stavrakakis, G. 2003. Motion on the Kaparelli fault (Greece) prior to the 1981 earthquake sequence determined from ^36^Cl cosmogenic dating. *Terra Nova,* **15,** 118–124.
7. Bernard, P., Lyon-Caen, H., Briole, P., Deschamps, A., Boudin, F., Makropoulos, K., Papadimitriou, P., Lemeille, F., Patau, G., Billiris, H., Paradissis, D., Papazissi, K., Castarède, H., Charade, O., Nercessian, A., Avallone, A., Pacchiani, F., Zahradnik, J., Sacks, S., & Linde, A. 2006. Seismicity, deformation and seismic hazard in the western rift of Corinth: New insights from the Corinth Rift Laboratory (CRL). *Tectonophysics,* **426,** 7-30.
8. Boccaletti, M., Caputo, R., Mountrakis, D., Pavlides, S., & Zouros, N. 1997. Paleoseismicity of the Souli Fault, Epirus, western Greece. *Journal of Geodynamics,* **24,** 117-127.
9. Bonatis, P., Karakostas, V., Kaplon, J., Papadimitriou, E., Kaviris, G., Ilieva, M., Foumelis, M., & Pikridis, C. 2024. Co-seismic and post-seismic slip associated with the 2021 M_w_5.9 Arkalochori, Central Crete (Greece) earthquake constrained by geodetic data and aftershocks. *Tectonophysics,* **22,** 230481. <https://doi.org/10.1016/j.tecto.2024.230481>.
10. Bornovas, J., & Rondogianni-Tsiambaou, Th. 1983. Geological map of Greece, 1:500.000. Institute of Geology and Mineral Exploration, Athens, Greece.
11. Cal, C., Boulton, S., & Mildon, Z. 2024. Structural and geomorphological constraints on the activity of the Sparta Fault (Greece). *Journal of the Geological Society of London,* **181,** jgs2024-066. <http://doi.org/10.1144/jgs2024-066>.
12. Caputo, R. 1993. Morphotectonics and kinematics of the Tyrnavos Fault, northern Larissa Plain, Greece. *Z. Geomorph. N.F.,* **94,** 167-185.
13. Caputo, R., & Pavlides, S. 1993. Late Cainozoic geodynamic evolution of Thessaly and surroundings (central-northern Greece). *Tectonophysics,* **223,** 339-662.
14. Caputo, R. 1995. Inference of a seismic gap from geological data: Thessaly (Central Greece) as a case study. *Annali di Geofisica,* **38,** 1-19. doi:10.4401/ag‐4127.
15. Caputo, R., & Helly, B. 2005. The Holocene activity of the Rodia Fault, Central Greece. *Journal of Geodynamics,* **40,** 153–169.
16. Caputo, R. 1996. The active Nea Anchialos Fault System (Central Greece): comparison of geological, morphotectonic, archaeological and seismological data. *Annali di Geofisica*, **39,** 557-574.
17. Caputo, R., Hinzen, K., Liberatore, D., Schreiber, S., Helly, B., & Tziafalias, A. 2010. Quantitative archaeoseismological investigation of the Great Theatre of Larissa, Greece. *Bulletin of Earthquake Engineering,* DOI 10.1007/s10518-010-9206-6.
18. Caputo, R., Chatzipetros, A., Pavlides, S., & Sboras, S. 2012. The Greek Database of Seismogenic Sources (GreDaSS): state-of-the-art for northern Greece. *Annals of Geophysics*, **55,** 859-894. doi: 10.4401/ag-5168.
19. Chatzipetros, A., Kokkalas, S., Pavlides, S., & Koukouvelas, I. 2005. Palaeoseismic data and their implication for active deformation in Greece. *Journal of Geodynamics,* **40,** 170–188.
20. Chatzipetros, A., Kiratzi, A., Sboras, S., Zouros, N., & Pavlides, S. 2013. Active faulting in the north-eastern Aegean Sea Islands. *Tectonophysics*, **597-598,** 106-122 106-122. <http://dx.doi.org/10.1016/j.tecto.2012.11.026>.
21. Chatzipetros, A., Pavlides, S., Foumelis, M., Sboras, S., Galanakis, D., Pikridas, C., Bitharis, S., Kremastas, E., Chatziioannou, A., & Papaioannou, I. (2021). The northern Thessaly strong earthquakes of March 3 and 4, 2021, and their neotectonic setting. *Bulletin of the Geological Society of Greece,* ***58*,** 222-255. doi:https://doi.org/10.12681/bgsg.27225
22. Collier, R., Pantosti, D., D’Addezio, G., De Martini, P., Masana, E., & Sakellariou, D. 1998. Paleoseismicity of the 1981 Corinth earthquake fault: Seismic contribution to extensional strain in central Greece and implications for seismic hazard. *Journal of Geophysical Research,* **103,** 30001-30019.
23. Cooper, F., Roberts, G., & Underwood, C. 2007. A comparison of 103–105 year uplift rates on the South Alkyonides Fault, central Greece: Holocene climate stability and the formation of coastal notches. *Geophysical Research Letters,* **34,** L14310. d oi:10.1029/2007GL030673.
24. Copley, A., Grutzner, C., Howell, A., Jackson, J., Penney, C., & Wimpenny, S. 2018. Unexpected earthquake hazard revealed by Holocene rupture on the Kenchreai Fault (central Greece): Implications for weak sub-fault shear zones. *Earth and Planetary Science Letters*, **486,** 141–154.
25. Cundy, A.B.; Kortekaas, S.; Dewez, T.; Stewart, I.S.; Collins, P.E.F.; Croudace, I.W.; Maroukian, H.; Papanastassiou, D.; Gaki-Papanastassiou, P.; Pavlopoulos, K.; & Dawson, A. 2000. Coastal wetlands as recorders of earthquake subsidence in the Aegean: a case study of the 1894 Gulf of Atalanti earthquakes, central Greece. *Marine Geology*, **170,** 3-26.
26. De Martini, P.M., Pantosti, D., Palyvos, N., Lemeille, F., McNeill, L., & Collier, R. 2004. Slip rates of the Aigion and Eliki Faults from uplifted marine terraces, Corinth Gulf, Greece. *Geoscience*, **336,** 325–334.
27. De Novellis, V., Reale, D., Adinolfi, G.M., Sansosti, E., & Convertito, V. (2021). Geodetic Model of the March 2021 Thessaly Seismic Sequence Inferred from Seismological and InSAR Data. *Remote Sensing*, 13, 3410. https://doi.org/10.3390/rs13173410
28. Deligiannakis, G., I., Papanikolaou, D., & Roberts, G. 2018. Fault specific GIS based seismic hazard maps for the Attica region, Greece. *Geomorphology*, **306,** 264–282, doi: 10.1016/j.geomorph.2016.12.005.
29. Feng, L.; Newman, A.V.; Farmer, G.T.; Psimoulis, P.; & Stiros, S. 2010. Energetic rupture, coseismic and post-seismic response of the 2008 Mw6.4 Achaia-Elia Earthquake in northwestern Peleponnese, Greece: an indicator of an immature transform fault zone. *Geophysical Journal International*, **183,** 103-110. doi: 10.1111/j.1365-246X.2010.04747.x.
30. Ford, M.; Rohais, S.; Williams, E.A.; Bourlange, S.; Jousselin, D.; Backert, N.; & Malartre, F. 2012. Tectono-sedimentary evolution of the western Corinth Rift (Central Greece). *Basin Research*, **25,** 3-25. doi: 10.1111/j.1365-2117.2012.00550.x
31. Fountoulis, I., Mariolakis, I., & Ladas, I. 2014. Quaternary basin sedimentation and geodynamics in SW Peleponnese (Greece) and late stage uplift of Taygetos Mt. *Bollettino di Geofisica Teorica ed Applicata*, **55,** 303-324. DOI 10.4430/bgta0074.
32. Gaki-Papanastassiou, K.; Maroukian, H.; & Papanastassiou, D. 2001. Recent coastal changes in the northern Laconian Gulf, Greece, based on geomorphological and archaeological evidence. *36th CIESM Congress Proceedings*, Monaco.
33. Gaki-Papanastasiou, K.; Karymbalis, E.; Papanastassiou, D.; & Maroukian, H. 2009. Quaternary marine terraces as indicators of neotectonic activity of the Ierapetra normal fault SE Crete (Greece). *Geomorphology,* **104,** 38-46.
34. Galanakis, D. 2001. Brittle tectonic and morphological alteration of Almyros basin. *Bulletin of the Geological Society of Greece,* **34,** 371-379. <http://dx.doi.org/10.12681/bgsg.17038>.
35. Galanakis, D., Sboras, S., Konstantopoulou, G., & Xenakis, M. (2021). Neogene-Quaternary tectonic regime and macroseismic observations in the Tyrnavos-Elassona broader epicentral area of the March 2021, intense earthquake sequence. *Bulletin of the Geological Society of Greece,* 58, 200-221. doi: <https://doi.org/10.12681/bgsg.27196>
36. Gallen, S.F.; Wegmann, K.W.; Bohnenstiehl, D.R.; Pazzaglia, F.J.; Brandon, M.T.; & Fassoulas, C. 2014. Active simultaneous uplift and margin-normal extension in a forearc high, Crete, Greece. *Earth and Planetary Science Letters*, **398,** 11-24. <http://dx.doi.org/10.1016/j.epsl.2014.04.038>.
37. Ganas, A., Pavlides, S. B., Sboras, S., Valkaniotis, S., Papaioannou, S., Alexandris, G. A., lessa, A., & Papadopoulos, G. A. 2004. Active fault geometry and kinematics in Parnitha Mountain, Attica, Greece. *Journal of Structural Geology*, **26,** 2103–2118.
38. Ganas, A., Pavlides, S., & Karastathis, V. 2005. DEM-based morphometry of range-front escarpments in Attica, central Greece, and its relation to fault slip rates. *Geomorphology*, **65,** 301–319.
39. Ganas, A.; Bosy, J.; Petro, L.; Drakatos, G.; Kontny, B.; Stercz, M.; Melis, N.; Cacon, S.; & Kiratzi, A. 2007. Monitoring active structures in eastern Corinth Gulf (Greece): the Kaparelli Fault. *Acta Geodynamica et Geomaterialia*, **4,** 67-75.
40. Ganas, A., Valkaniotis, S., Briole, P., Serpetsidaki, A., Kapetanidis, V., Karasante, I., Kassaras, I., Papathanassiou, G., Karamitros, I., Tsironi, V., Elias, P., Sarhosis, V., Karakonstantis, A., Konstantakopoulou, E., Papadimitriou, P., & Sokos, E. 2021. Domino-style earthquakes along blind normal faults in Northern Thessaly (Greece): kinematic evidence from field observations, seismology, SAR interferometry and GNSS. *Bulletin of the Geological Society of Greece,* **58,** 37-86. <https://doi.org/10.12681/bgsg.27102>.
41. Ganas, A., Valkaniotis, S., Briole, P., Serpetsidaki, A., Kapetanidis, V., Karasante, I., Kassaras, I., Papathanassiou, G., Karamitros, I., Tsironi, V., Elias, P., Sarhosis, V., Karakonstantis, A., Konstantakopoulou, E., Papadimitriou, P., & Sokos, E. (2021). Domino-style earthquakes along blind normal faults in Northern Thessaly (Greece): kinematic evidence from field observations, seismology, SAR interferometry and GNSS. *Bulletin of the Geological Society of Greece,* ***58*,** 37-86. doi: https://doi.org/10.12681/bgsg.27102
42. Gawthorpe, R.L., Leeder, M.R., Kranis, H., Skourtsos, E., Andrews, J.E., Henstra, G.A., Mack, G.H., Muravchik, M., Turner, J.A., Stamatakis, M. 2017. Tectono-sedimentary evolution of the Plio-Pleistocene Corinth rift, Greece. *Basin Research*, **30,** 448-479. doi: 10.1111/bre.12260.
43. Ghisetti, F., & Vezzani, L. 2005. Inherited structural controls on normal fault architecture in the Gulf of Corinth (Greece). *Tectonics*, **24,** TC4016. doi:10.1029/2004TC001696.
44. Goldsworthy, M., & Jackson, J. 2000. Active normal fault evolution in Greece revealed by geomorphology and drainage patterns. *Journal of the Geological Society of London*, **157,** 967–981.
45. Goldsworthy, M., & Jackson, J. 2001. Migration of activity within normal fault systems: examples from the Quaternary of mainland Greece. *Journal of Structural Geology*, **23,** 489-506.
46. Goldsworthy, M., Jackson, J., & Haines, J. 2002. Continuity of active fault systems in Greece, *Geophysical Journal International,* **148,** 596–618.
47. Gouliotis, L.; & Papanikolaou, D. 2024. The Northern Giona Fault Zone, a Major Active Structure Through Central Greece. *GeoHazards*, **5,** 1370-1388. <https://doi.org/10.3390/geohazards5040065>.
48. Grützner, C., Schneiderwind, S., Papanikolaou, I., Deligiannakis, G., Pallikarakis, A., & Reicherter, K. 2016. New constraints on extensional tectonics and seismic hazard in northern Attica, Greece—The case of the Milesi fault. *Geophysical Journal International*, **204,** 180–199, doi:10.1093/gji/ggv443.Iezzi et al. 2021.
49. Jackson J.A.; Gagnepain, J.; Houseman, G.; King, G.C.P.; Papadimitriou, P.; Sourfleris, C.; & Virieux, J. 1982. Seismicity, normal faulting, and the geomorphological development of the Gulf of Corinth (Greece): the Corinth earthquakes of February and March 1981. *Earth and Planetary Science Letters*, **57,** 377-397.
50. Karakitsios, V. 2013. Western Greece and Ionian Sea petroleum systems. *American Association of Petroleum Geologists*, **97,** 1567-1595. DOI: 10.1306/02221312113.
51. Karakostas, V., Papazachos, C., Papadimitriou, E., Foumelis, M., Kiratzi, A., Pikridas, C., Kostoglou, A., Kkallas, C., Chatzis, N., Bitharis, S., Chatzipetros, A., Fotiou, A., Ventouzi, C., Karagianni, E., Bonatis, P., Kourouklas, C., Paradisopoulou, P., Scordilis, E., Vamvakaris, D., Grendas, I., Kementzetzidou, D., Panou, A., Karakaisis, G., Karagianni, I., Hatzidimitriou, P., & Galanis, O. (2021). The March 2021 Tyrnavos, central Greece, doublet (Μw6.3 and Mw6.0): Aftershock relocation, faulting details, coseismic slip and deformation. *Bulletin of the Geological Society of Greece,* ***58*,** 131-178. doi:https://doi.org/10.12681/bgsg.27237
52. Karamitros, I., Ganas, A., Chatzipetros, A., & Valkaniotis, S. 2020. Non-planarity, scale-dependent roughness and kinematic properties of the Pidima active normal fault scarp (Messinia, Greece) using high-resolution terrestrial LiDAR data. *Journal of Structural Geology*, **136,** 104065.
53. Kassaras, I., Kapetanidis, V., Ganas, A., Karakonstantis, A., Papadimitriou, P., Kaviris, G., Kouskouna, V., & Voulgaris, N. (2022). Seismotectonic analysis of the 2021 Damasi-Tyrnavos (Thessaly, Central Greece) earthquake sequence and implications on the stress field rotations. *Journal of Geodynamics,* **150,** 101898, doi.org/10.1016/j.jog.2022.101898.
54. Kilias, A., Falalakis, G., Sfeikos, A., Papadimitriou, E., Vamvaka, A., & Gkarlaouni, C. 2013. The Thrace basin in the Rhodope province of NE Greece — A tertiary supradetachment basin and its geodynamic implications. *Tectonophysics,* **595-596,** 90-105.
55. Kokkalas, S., Jones, R., McCaffery, K., & Clegg, P. 2007. Quantitative fault analysis at Arkitsa, Central Greece, using terrestrial laser-scanning (“LiDAR”). *Bulletin of the Geological Society of Greece vol. XXXVII, 2007*, Proceedings of the 11th International Congress, Athens, May, 2007.
56. Kokkalas, S., Pavlides, S., Koukouvelas, I., Ganas, A., & Stamatopoulos, L. 2007. Paleoseismicity of the Kaparelli fault (eastern Corinth Gulf): evidence for earthquake recurrence and fault behavior. *Boll. Soc. Geol. It.,* **126,** 387-395.
57. Konstantinou, K.I., Melis, N.S., Lee, S.-J., Evangelidis, C.P., Boukouras, K., 2009. Rupture process and aftershocks relocation of the 8 June 2008 Mw 6.4 earthquake in Northwest Peloponnese, Western Greece. Bull. Seismol. Soc. Am. **99**, 3374–3389.
58. Konstantinou, K., Mouslopoulou, V., & Saltogianni, V. 2020. Seismicity and Active Faulting around the Metropolitan Area of Athens, Greece. *Bulletin of the Seismological Society of America*, **XX,** 1–18. doi: 10.1785/0120200039.
59. Koukouvelas, I. 1998. The Egion Fault, earthquake-related and long-term deformation, Gulf of Corinth, Greece. *Journal of Geodynamics*, **26,** 501-513.
60. Koukouvelas, I., & Aydin, A. 2002. Fault structure and related basins of the North Aegean Sea and its surroundings. *Tectonics*, **21,** 10.1029/2001TC901037.
61. Koukouvelas, I., Kokkalas, S., & Xypolias, P. 2009. Surface deformation during the Mw 6.4 (8 June 2008) Movri Mountain earthquake in the Peloponnese, and its implications for the seismotectonics of western Greece. *International Geology Review*, DOI: 10.1080/00206810802674329.
62. Koukouvelas, I., Zygouri, V., Papadopoulos, A., & Verroios, S. 2017. Holocene record of slip-predictable earthquakes on the Kenchreai Fault, Gulf of Corinth, Greece. *Journal of Structural Geology*, **94,** 258-274.
63. Koukouvelas, I., Piper, D., Katsonopoulou, D., Kontopoulos, N., Verroios, S., Nikolakopoulos, K., & Zygouri, V. 2020. Earthquake-triggered landslides and mudflows: Was this the wave that engulfed Ancient Helike? *The Holocene,* 1-16. DOI: 10.1177/0959683620950389.
64. Koukouvelas, I.K., Nikolakopoulos, K.G., Kyriou, A., Caputo, R., Belesis, A., Zygouri, V., Verroios, S., Apostolopoulos, D., & Tsentzos, I. 2021. The March 2021 Damasi Earthquake Sequence, Central Greece: Reactivation Evidence across the Westward Propagating Tyrnavos Graben. *Geosciences*, **11,** 328. <https://doi.org/10.3390/geosciences11080328>.
65. Koukouvelas, I. K., Caputo, R., Nikolakopoulos, K. G., Kyriou, A., & Famiglietti, N. A. (2023). Is the Mesochori Fault a Key Structure for Understanding the Earthquake Activity during the 2021 Damasi Earthquakes in Northern Thessaly, Greece? *Geosciences*, ***13***, 331. <https://doi.org/10.3390/geosciences13110331>
66. Lazos, I., Papanikolaou, I., Sboras, S., Foumelis, M., & Pikridas, C. 2022. Geodetic Upper Crust Deformation Based on Primary GNSS and INSAR Data in the Strymon Basin, Northern Greece—Correlation with Active Faults. *Applied Sciences*, ***12***, 9391.
67. Leeder, M.R., Collier, R.E.Ll., Abdul Aziz, L.H., Trout, M., Ferentinos, G., Papatheodorou, & G., Lyberis, E. 2002. Tectono-sedimentary processes along an active marine/lacustrine half-graben: Alkyonides Gulf, E. Gulf of Corinth, Greece. *Basin Research* **14,** 25-41.
68. Lekkas, E., D. Papanikolaou, and I. Fountoulis (1992). Neotectonic Map of Greece, Scale 1:100, 000, *Department of Dynamic, Tectonic and Applied Geology, University of Athens*, Athens, Greece.
69. Lekkas, E., Papanikolaou, D., & Sakellariou, D. 1993. Neotectonic map of Greece, Rhodes Island (scale 1:100.000). *Research Project, University of Athens,* 99p., Athens.
70. Lekkas, E., Papanikolaou, D., Lozios, S., Papoulia, I., & Vasilopoulou, S. 1995. Neotectonic Map of Eastern Attica, *Department of Dynamic, Tectonic and Applied Geology (University of Athens) & Prefecture of Eastern Attica,* Athens, Greece.
71. Lekkas, E. 1996. Neotectonic map of Greece. Cephalonia sheet. Scale 1:1,000,000. *University of Athens,* 144 p.
72. Lekkas, E., Lozios, S., Skourtsos, E., & Kranis, H. 1998. Egio Earthquake (15 June 1995): An episode in the neotectonic evolution of Corinthiakos Gulf. *Journal of Geodynamics*, **26,** 487-499.
73. Lekkas, E., Danamos, G., & Lozios, S. 2001. Neotectonic structure and neotectonic evolution of Lefkada Island. *Bulletin of the Geological Society of Greece*, **XXXIV/I,** 157-163.
74. Lyon-Caen, H., Armijo, R., Drakopoulos, J., Baskoutass, J., Delibassis, N., Gaulon, R., Kouskouna, V., Latoussakis, J., Makropoulos, K., Papadimitriou, P., Papanastassiou, D., & Pedotti, G. 1988. The 1986 Kalamata ( South Peloponnesus) Earthquake: Detailed Study of a Normal Fault, Evidences for East-West Extension in the Hellenic Arc. *Journal of Geophysical Research*, **93,** 14967-15000.
75. Mack, G., Leeder, M., & Perez-Arlucea, M. 2009. Late Neogene rift-basin evolution and its relation to normal fault history and climate change along the southwestern margin of the Gerania Range, central Greece. *Geological Society of America Bulletin*, **121,** 907-918. doi:10.1130/B26337.1.
76. Manta, K., Rousakis, G.; Anastasakis, G., Lykousis, V., Sakellariou, D., Panagiotopoulos, I. P. 2019. Sediment transport mechanisms from the slopes and canyons to the deep basins south of Crete Island (southeast Mediterranean). *Geo-Marine Letters*, **39,** DOI:10.1007/s00367-019-00575-1.
77. Mariolakos, I., Fountoulis, I., Logos, E., & Lozios, S. 1989. Surface faulting caused by the Kalamata (Greece) earthquakes (13.9.86). *Tectonophysics*, **163,** 197-203. <https://doi.org/10.1016/0040-1951(89)90257-6>.
78. Mariolakos, I., Fountoulis, I., Mariolakos, D., Andreadakis, M., & Georgakopoulos, A. 2000. Geodynamic phenomena observed during the Athens earthquake (Ms=5.9) 7-9-99. *Annales Geologiques des Pays Helleniques*, **38,** 175-186.
79. Mariolakos, H., Fountoulis, I., & Ladas, I. 2001. Paleogeographic Evolution of SW Peloponnesus during Quaternary. *Bulletin of the Geological Society of Greece,* **34,** 37-45. <http://dx.doi.org/10.12681/bgsg.16941>.
80. Mason, J., Schneiderwind, S., Pallikarakis, A., Wiatr, T., Mechernich, S., Papanikolaou, & I., Reicherter, K. 2016. Fault structure and deformation rates at the Lastros-Sfaka Graben, Crete. *Tectonophysics* **683,** 216–232.
81. Mechernich, S., Schneiderwind, S., Mason, J., Papanikolaou, I., Deligiannakis, G., Pallikarakis, A., Binnie, S., Dunai, T., & Reicherter, K. 2018. The seismic history of the Pisia fault (eastern Corinth rift, Greece) from fault plane weathering features and cosmogenic 36Cl dating. *Journal of Geophysical Research: Solid Earth,* **123.** <https://doi.org/10.1029/2017JB014600>.
82. Mechernich, S., Reicherter, K., Deligiannakis, G., & Papanikolaou, I. 2022. Tectonic geomorphology of active faults in Eastern Crete (Greece) with slip rates and earthquake history from cosmogenic 36Cl dating of the Lastros and Orno faults. *Quaternary International.* doi.org/10.1016/j.quaint.2022.04.007.
83. Michailidou A., Chatzipetros A., & Pavlides S. 2005. Quantitative analysis - tectonic geomorphology indicators of the faults at the region of Stratoni-Varvara Gomati-M. Panagia in the eastern Chalkidiki. *Bulletin of the Geological Society of Greece,* **38,** 14-29.
84. Morewood, N.C., & Roberts, G.P. 1999. Lateral propagation of the surface trace of the South Alkyonides normal fault segment, central Greece: its impact on models of fault growth and displacement-length relationships. *Journal of Structural Geology*, **21,** 635-652.
85. Morewood, N.C., & Roberts, G.P. 2002. Surface observations of active normal fault propagation: implications for growth. *Journal of the Geological Society, London*, **159,** 263-272.
86. Mountrakis, D., Kilias, A., Pavlides, S., Sotiriadis, L., Psilovikos, A., Astaras Th, V. E., & Skordilis, M. 1996. Neotectonic map of Greece, Langadhas sheet. *Earthquake planning and protection organisation and European centre on prevention and forecasting of earthquakes.* Scale, 1:100,000.
87. Mountrakis, D., Tranos, M., Papazachos, C., Thomaidou, E., Karagianni, E., & Vamvakaris, D. 2006. Neotectonic and seismological data concerning major active faults, and the stress regimes of Northern Greece. *Geological Society, London, Special Publications,* **260,** 649–670.
88. Mountrakis, D., Kilias, A., Pavlaki, A., Fassoulas, C., Thomaidou, E., Papazachos, C., Papaioannou, C., Roumelioti, Z., Benetatos, C., & Vamvakaris, D. 2012. Neotectonic study of Western Crete and implications for seismic hazard assessment. In: (Eds.) Emmanuel Skourtsos and Gordon S. Lister, *Journal of the Virtual Explorer*, **42,** 2, doi:10.3809/jvirtex.2011.00285
89. Mountrakis, D., Kilias, A., Pavlaki, A., Fassoulas, C., Thomaidou E., Papazachos, C., Papaioannou, C., Roumelioti, Z., Benetatos, C., & Vamvakaris, D. 2013. Neotectonic analysis, active stress field and active faults seismic assessment in western Crete. *Bulletin of the Geological Society of Greece,* **XLVII**, Proceedings of the 13th International Congress, Chania, Sept. 2013.
90. Mouslopoulou, V., Andreou, C., Atakan, K., & Fountoulis, I. 2001. Paleoseismological investigations along the Kera fault zone, Western Crete: implications for seismic hazard assessment. *Bulletin of the Geological Society of Greece,* **34,** 1531-1537. <https://doi.org/10.12681/bgsg.17259>.
91. Mouslopoulou, V., Moraetis, D., Benedetti, L., Guillou, V., Bellier, O., & Hristopulos, D. 2014a. Normal faulting in the forearc of the Hellenic subduction margin: Paleoearthquake history and kinematics of the Spili Fault, Crete, Greece. *Journal of Structural Geology*, ***66*,** 298–308.
92. Mouslopoulou, V., Saltogianni, V., Gianniou, M., & Stiros, S. 2014b. Geodetic evidence for tectonic activity on the Strymon Fault System, northeast Greece. *Tectonophysics,* **633**, 246-255,  <https://doi.org/10.1016/j.tecto.2014.07.012>
93. Mouslopoulou, V., Bocchini, G.-M., Cesca, S., Saltogianni, V., Bedford, J., Petersen, G., Gianniou, M., & Oncken, O. (2020). Earthquake swarms, slow slip, and fault interactions at the western end of the Hellenic Subduction System precede the Mw 6.9 Zakynthos Earthquake, Greece. *Geochemistry, Geophysics, Geosystems*, **21,** e2020GC009243. <https://doi.org/10.1029/2020GC009243>.
94. Mouslopoulou, V., Sudhaus, H., Konstantinou, K., Begg, J., Saltogianni, V., Maennel, B., Andinisari, R., & Oncken, O. 2022. A deeper look into the 2021 Tyrnavos Earthquake sequence (TES) reveals coseismic breaching of an unrecognized large‐scale fault relay zone in continental Greece. *Tectonics*, **41**, DOI: 10.1029/2022TC007453.
95. Nance, D. 2010. Neogene-Recent extension on the eastern flank of Mount Olympus, Greece. *Tectonophysics*, **488**, 282-292, doi:10.1016/j.tecto.2009.05.011.
96. Nicol, A., Mouslopoulou, V., Begg, J., & Oncken, O. 2020. Displacement accumulation and sampling of paleoearthquakes on active normal faults of Crete in the eastern Mediterranean. *Geochemistry, Geophysics, Geosystems,* **21,** e2020GC009265, doi: 10.1029/2020GC009265.
97. Ntokos, D. 2017. Synthesis of literature and field work data leading to the compilation of a new geological map—A review of geology of Northwestern Greece. *International Journal of Geosciences,* **8,** 205-236. doi: 10.4236/ijg.2017.82009.
98. Ntokos, D. 2018. Neotectonic study of Northwestern Greece. *Journal of Maps,* **14,** 178–188. <https://doi.org/10.1080/17445647.2018.1445562>.
99. Ntokos, D. 2021. Age estimation of tectonically exposed surfaces using cation-ratio dating of rock varnish. *Catena,* **200***,* 105167. doi.org/10.1016/j.catena.2021.105167.
100. Palyvos, N., Pantosti, D. De Martini, P.M. Lemeille, F. Sorel, D., & Pavlopoulos, K. 2005. The Aigion – Neos Erineos coastal normal fault system (western Corinth Gulf Rift, Greece): Geomorphological signature, recent earthquake history, and evolution, *Journal of Geophysical Research,* **110,** B09302, doi:10.1029/2004JB003165.
101. Palyvos, N., Bantekas, I., Kranis, H. 2006. Transverse fault zones of subtle geomorphic signature in northern Evia Island (central Greece extensional province): An introduction to the Quaternary Nileas graben. *Geomorphology*, **76,** 363-374.
102. Palyvos, N., Pavlopoulos, K. Froussou, E. Kranis, H. Pustovoytov, K. Forman, S. L., & Minos‐Minopoulos D. 2010. Paleoseismological investigation of the oblique‐normal Ekkara ground rupture zone accompanying theM6.7–7.0 earthquake on30 April 1954 in Thessaly, Greece: Archaeological and geochronological constraints on ground rupture recurrence. *Journal of Geophysical Research*, **115,** B06301, doi:10.1029/2009JB006374.
103. Pantosti, D., Collier, R., D’Addezio, G., Masana, E., Sakellariou, D. 1996. Direct geological evidence for prior earthquakes on the 1981 Corinth fault (central Greece). *Geophysical Research Letters,* **23**, 3795-3798.
104. Pantosti, D., De Martini, P., Koukouvelas, I., Stamatopoulos, L., Palyvos, N., Pucci, S., Lemeille, F., Pavlides, S. 2004. Palaeoseismological investigations of the Aigion Fault (Gulf of Corinth, Greece). *C. R. Geoscience,* **336,** 335–342.
105. Papadopoulos, G., Pavlides, S. 1992. The large 1956 earthquake in the South Aegean: Macroseismic field configuration, faulting, and neotectonics of Amorgos Island. *Earth and Planetary Science Letters,* **113,** 383-396.
106. Papanikolaou, D., Lozios, S., Sideris, C., Kranis, H., Danamos, G., Soukis, K., Skourtsos, E., Bassi, E., Marinos, P., Tsiampaos, et al. 2002. Geological–Geotechnical study of Athens basin, in OASP Applied Research Program, OASP, Athens, Greece, 152 pp.Papanikolaou, D., and I. Papanikolaou (2007). Geological, geomorphological and tectonic structure of NE Attica and seismic hazard implications for the northern edge of the Athens plain, *Bulletin of the Geological Society of Greece,* **40,** 425–438, doi: 10.12681/bgsg.16634.
107. Papanikolaou, D., Papanikolaou, I.D. 2007. Geological, Geomorphological and tectonic structure of NE Attika and seismic hazard implications for the northern edge of the Athens Plain. Bulletin of the Geological Society of Greece , **XXXX,** 425-438.
108. Papanikolaou, D., Fountoulis, I., Metaxas, Ch. 2007. Active faults, deformation rates and Quaternary paleogeography at Kyparissiakos Gulf (SW Greece) deduced from onshore and offshore data. Quaternary International, **171,** 14-30.
109. Papanikolaou, I., Papanikolaou, D., Lekkas, E. 2008. Low slip-rate faults around big cities: A challenging threat. The Afindai Fault as a case study for the city of Athens. The 14th World Conference on Earthquake Engineering October 12-17, 2008, Beijing, China.
110. Papanikolaou, I., Roberts, G., Deligiannakis, G., Sakellariou, D., Vassilakis, E. 2013. The Sparta Fault, Southern Greece: From segmentation and tectonic geomorphology to seismic hazard mapping and time dependent probabilities. *Tectonophysics*, **597–598,** 85–105.
111. Papanikolaou, I., Triantaphyllou, M., Pallikarakis, A., Migiros, G. 2015. Active faulting at the Corinth Canal based on surface observations, borehole data and paleoenvironmental interpretations. Passive rupture during the 1981 earthquake sequence? *Geomorphology*, **237,** 65–78.
112. Papanikolaou, I., Dafnis, P., Deligiannakis, G., Hengesh, J., Panagopoulos, A. 2022. Active faults, Paleoseismological trenching and seismic hazard assessment in the Northern Mygdonia Basin, Northern Greece: The Assiros-Krithia fault and the Drimos fault zone. *Quaternary International*, **651,** 92-107.
113. Pavlides, S.B., & Tranos, M.D. 1991. Structural characteristics of two strong earthquakes in the North Aegean: Ierissos (1932) and Agios Efstratios (1968). *Journal of Structural Geology*, **13,** 205–214.
114. Pavlides, S. 1996. First Palaeoseismological results from Greece. *Annali di Geofisica*, **34,** 545–555.
115. Pavlides, S., Chatzipetros, A., Sboras, S., Ganas, A., Papathanassiou, G., Valkaniotis, S. 2008. Identification of active tectonic structures using geologic data. Report included in the final report of the project “Contribution of advanced methods of Geosciences in seismic risk management with emphasis to the built environment of the North Aegean Sea Islands,” funded by General Secretariat of Research and Technology, [PEP–NAEGEAN], 2006–31.05.2008.
116. Pavlides, S. B., & Sboras, S. P. 2021. Recent earthquake activity of March 2021 in northern Thessaly unlocks new scepticism on Faults. *Turkish Journal of Earth Sciences*, *30*(SI-1), 851–861. <https://doi.org/10.3906/yer-2110-6>
117. Perouse, E., Sebrier, M., Braucher, R., Chamot-Rooke, N., Bourles, D., Briole, P., Sorel, D., Dimitrov, D., Arsenikos, S. 2016. Transition from collision to subduction in Western Greece: the Katouna–Stamna active fault system and regional kinematics. *International Journal of Earth Sciences*, **106,** 967–989. <https://doi.org/10.1007/s00531-016-1345-9>.
118. Peterek, A., & Schwarze, J. 2004. Architecture and Late Pliocene to recent evolution of outer-arc basins of the Hellenic subduction zone (south-central Crete, Greece). *Journal of Geodynamics*, **38,** 19–55.
119. Roberts, G., & Ganas, A. 2000. Fault‐slip directions in central and southern Greece measured from striated and corrugated fault planes: Comparison with focal mechanism and geodetic data. *Journal of Geophysical Research: Solid Earth*, **105**, 23443–23462.
120. Robertson, J., Roberts, G., Iezzi, F., Meschis, M., Gheorghiu, D., Sahy, D., Bristow, C., & Sgambato, C. 2020. Distributed normal faulting in the tip zone of the South Alkyonides Fault System, Gulf of Corinth, constrained using 36Cl exposure dating of late-Quaternary wave-cut platforms. *Journal of Structural Geology*, **136,** 104063.
121. Rodriguez, M., Sakellariou, D., Gorini, C., Janin, A., D’Acremont, E., Le Pourhiet, L., Chamot-Rooke, N., Tsampouraki-Kraounaki, K., Morfis, I., Rousakis, G., Henry, P., Lurin, A., Delescluse, M., Briole, P., Rigo, A., Arsenikos, S., Bulois, C., Fernandez-Blanco, D., Beniest, A., Grall, C., Chanier, F., Caroir, F., Dessa, J.-X., Oregioni, D., Nercessian, A. 2023. Evolution of the North Anatolian Fault from a diffuse to a localized shear zone in the North Aegean Sea during the Plio-Pleistocene. *Geophysical Journal International* **235,** 2614–2639. <https://doi.org/10.1093/gji/ggad364>.
122. Sakellariou, D., Lykousis, V., Alexandri, S., Kaberi, H., Rousakis, G., Nomikou, P., Georgiou, P., & Ballas, D. 2007. Faulting, seismic, stratigraphic architecture and late Quaternary evolution of the Gulf of Alkyonides Basin–East Gulf of Corinth, central Greece. *Basin Research* **19,** 273–295.
123. Sboras, S., Ganas, A., & Pavlides, S. 2010. Morphotectonic analysis of the neotectonic and active faults of Beotia (central Greece) using GIS techniques. *Bulletin of the Geological Society of Greece,* **43,** 1607–1618. <https://doi.org/10.12681/bgsg.11335>.
124. Sboras, S., Lazos, I., Mouzakiotis, E., Karastathis, V., & Pavlides, S. 2020. Fault modelling, seismic sequence evolution and stress transfer scenarios for the July 20, 2017 (Mw 6.6) Kos–Gökova Gulf earthquake, SE Aegean. *Acta Geophysica*. <https://doi.org/10.1007/s11600-020-00471-8>.
125. Skourtsos, E., & Lekkas, S. 2011. Extensional tectonics in Mt Parnon (Peloponnesus, Greece). *International Journal of Earth Sciences,* **100,** 1551–1567.
126. Spyrou, E., Triantaphyllou, M.V., Tsourou, T., Vassilakis, E., Asimakopoulos, C., Konsolaki, A., Markakis, D., Marketou-Galari, D., & Skentos, A. 2022. Assessment of Geological Heritage Sites and Their Significance for Geotouristic Exploitation: The Case of Lefkas, Meganisi, Kefalonia and Ithaki Islands, Ionian Sea, Greece. *Geosciences,* **12,** 55. <https://doi.org/10.3390/geosciences12020055>.
127. Stamatopoulos, E., Kamberis, E., & Alevizos, G. 2019. Detection of active fault scarps in Western Peloponnese, Greece. *Quaternary International,* **508,** 88–97.
128. ten Veen, J.H., & Kleinspehn, K.L. 2003. Incipient continental collision and plate-boundary curvature: Late Pliocene–Holocene transtenstional Hellenic forearc, Crete, Greece. *Journal of the Geological Society,* **160,** 1–21.
129. Tranos, M., Papadimitriou, E., & Kilias, A. 2003. Thessaloniki-Gerakarou Fault Zone (TGFZ): the western extension of the 1978 Thessaloniki earthquake fault (Northern Greece) and seismic hazard assessment. *Journal of Structural Geology,* **25,** 2109–2123.
130. Tranos, M.D., & Mountrakis, D.M. 2004. The Serres fault zone (SZF): an active fault zone in Eastern Macedonia (Northern Greece). *5th International Symposium on Eastern Mediterranean Geology*, Thessaloniki, Greece, 14–20 April 2004, Proceedings, S1–18.
131. Tranos, M., Meladiotis, I.D., & Tsolakopoulos, E.P. 2004. Geometrical characteristics, scaling properties and seismic behavior of the faulting of the Chortiatis region and Anthemountas Basin (Northern Greece). In: *5th International Symposium on Eastern Mediterranean Geology*, Thessaloniki, Greece, edited by A.A. Chatzipetros and S.B. Pavlides, **2,** 889–891.
132. Tranos, M., Kachev, V., & Mountrakis, D. 2008. Transtensional origin of the NE–SW Simitli basin along the Strouma (Strymon) Lineament, SW Bulgaria. *Journal of the Geological Society, London,* **165,** 499–510.
133. Tranos, M.D. 2011. Strymon and Strymonikos Gulf basins (Northern Greece): Implications on their formation and evolution from faulting. *Journal of Geodynamics,* **51,** 285–305. <https://doi.org/10.1016/j.jog.2010.10.002>.
134. Tsimi, C., Ganas, A., Soulakellis, N., Kairis, O., & Valmis, S. 2007. Morphotectonics of the Psathopyrgos active fault, western Corinth Rift, central Greece. *Bulletin of the Geological Society of Greece* XXXVII, Proceedings of the 11th International Congress, Athens, May 2007.
135. Tsironi, V., Ganas, A., Valkaniotis, S., Kouskouna, V., Sokos, E., & Koukouvelas, I. 2024. Active shortening and aseismic slip along the Cephalonia Plate Boundary (Paliki Peninsula, Greece): Evidence from InSAR and GNSS data. *Tectonophysics,* **884**, 230400. <https://doi.org/10.1016/j.tecto.2024.230400>.
136. Tsodoulos, I.M., Koukouvelas, I.K., & Pavlides, S. 2008. Tectonic geomorphology of the easternmost extension of the Gulf of Corinth (Beotia, Central Greece). *Tectonophysics,* **453,** 211–232.
137. Tsodoulos, I.M., Gallousi, C., Chatzipetros, A., Stamoulis, K., Ioannides, K., & Pavlides, S. 2015. A new paleoseismological investigation across the Paleochori–Sarakina Fault, Northern Greece: Trenching exposures of the surface rupture of 1995 Ms 6.6 Kozani–Grevena earthquake. *Geophysical Research Abstracts* 17, EGU2015-12891.
138. Tsodoulos, I.M., Stamoulis, K., Caputo, R., Koukouvelas, I., Chatzipetros, A., Pavlides, S., Gallousi, C., Papachristodoulou, C., & Ioannides, K. 2016. Middle–Late Holocene earthquake history of the Gyrtoni Fault, Central Greece: Insight from optically stimulated luminescence (OSL) dating and paleoseismology. *Tectonophysics,* **687,** 14–27. <https://doi.org/10.1016/j.tecto.2016.08.015>.
139. Tsodoulos, I.M., Gallousi, C., Stamoulis, K., Chatzipetros, A., Pavlides, S., & Ioannides, K. 2024. Tectonic geomorphology and paleoseismology of the Angelochori fault segment of the Anthemountas extensional detachment fault, Central Macedonia, Greece: Paleoseismic evidence from the 1677 CE earthquake. *Geomorphology*, 109372. <https://doi.org/10.1016/j.geomorph.2024.109372>.
140. Valkaniotis, S., & Pavlides, S. 2016. Late Quaternary and Holocene faults of the northern Gulf of Corinth Rift, Central Greece. *Bulletin of the Geological Society of Greece* L, 164–172. Proceedings of the 14th International Congress, Thessaloniki, May 2016.
141. Veliz, V., Mouslopoulou, V., Nicol, A., Fassoulas, C., Begg, J., & Oncken, O. 2018. Millennial to million-year normal-fault interactions on the forearc of a subduction margin, Crete, Greece. *Journal of Structural Geology,* **113,** 225–241. <https://doi.org/10.1016/j.jsg.2018.05.019>
142. Veliz-Borel, V., Mouslopoulou, V., Nicol, A., Begg, J., & Oncken, O. 2022. Normal faulting along the Kythira–Antikythira Strait, southwest Hellenic forearc, Greece. *Frontiers in Earth Science,* **9,** 730806. <https://doi.org/10.3389/feart.2021.730806>.
143. Veliz, V., Mouslopoulou, V., Glodny, J., Begg, J., Metzger, S., Sakellariou, D., & Oncken, O. 2024. Exploring uplift mechanisms across the forearc of a subduction system: Karpathos Island as a natural transect across the eastern Hellenic margin. Tectonics, **43,** e2023TC008156. <https://doi.org/10.1029/2023TC008156>
144. Wicker, V., Ford, M., Gawthorpe, R.L., Skourtsos, E., Kranis, H., Kerouedan, L., & Muravchik, M. 2024. Transition from Late‐Miocene syn‐orogenic extension to Plio‐Pleistocene Corinth rifting in the southern Hellenides, northern Peloponnese, Greece. *Tectonics,* **43,** e2023TC007964. <https://doi.org/10.1029/2023TC007964>.
145. Zelenin, E.A., Bachmanov, D.M., Garipova, S.T., Trifonov, V.G., & Kozhurin, A.I. 2022. The Active Faults of Eurasia Database (AFEAD): the ontology and design behind the continental-scale dataset. *Earth System Science Data,* **14,** 4489–4503.
146. Zervopoulou, A., & Pavlides, S. 2005. Morphotectonic study of the broader area of Thessaloniki for the cartography of neotectonic faults. *Bulletin of the Geological Society of Greece*, **XXXVIII,** 30–41.
147. Zervopoulou, A., & Pavlides, S. 2008. Νεοτεκτονικά ρήγματα πολεοδομικού συγκροτήματος Θεσσαλονίκης (Neotectonic faults in the urban site of Thessaloniki). *Πανελλήνιο Συνέδριο Αντισεισμικής Μηχανικής & Τεχνικής Σεισμολογίας*.
148. Zygouri, V., Koukouvelas, I., Kokkalas, S., Xypolias, P., & Papadopoulos, G. 2015. The Nisi Fault as a key structure for understanding the active deformation of the NW Peloponnese, Greece. *Geomorphology,* **237,** 142–156.
149. Zygouri, V., Koukouvelas, I., & Ganas, A. 2016. Palaeoseismological analysis of the east Giouchtas Fault, Heraklion Basin, Crete (Preliminary Results). *Bulletin of the Geological Society of Greece* L, 563–571. Proceedings of the 14th International Congress, Thessaloniki, May 2016.
150. Zygouri, V., Koukouvelas, I., Ganas, A., & Tsimi, C. 2023. Clustering of earthquakes along the Pidima–Anthia normal fault: New data from palaeoseismology and tectonic geomorphology and their significance on the earthquake’s regularity and recurrence across southern Greece. *Journal of Structural Geology*, **176,** 104974. <https://doi.org/10.1016/j.jsg.2023.104974>.
151. Walker, R.T., Claisse, S., Telfer, M., Nissen, E., England, P., Bryant, C., Bailey, R. 2010. Preliminary estimate of Holocene slip rate on active normal faults bounding the southern coast of the Gulf of Evia, central Greece. Geosphere **6,** 583–593. doi: <https://doi.org/10.1130/GES00542.1>
